# Consumer Preferences for Written and Oral Information about Allergens When Eating Out

**DOI:** 10.1371/journal.pone.0156073

**Published:** 2016-05-25

**Authors:** Fiona M. Begen, Julie Barnett, Ros Payne, Debbie Roy, M. Hazel Gowland, Jane S. Lucas

**Affiliations:** 1 Department of Psychology, University of Bath, Bath, United Kingdom; 2 Creative Research Ltd, Bishops Castle, United Kingdom; 3 Allergy Action, St. Albans, United Kingdom; 4 Clinical and Experimental Sciences, Faculty of Medicine, University of Southampton, Southampton, United Kingdom; University of Alabama at Birmingham, UNITED STATES

## Abstract

**Background:**

Avoiding food allergens when eating outside the home presents particular difficulties for food allergic (FA) and intolerant (FI) consumers and a lack of allergen information in restaurants and takeaways causes unnecessary restrictions. Across Europe, legislation effective from December 2014, aims to improve allergen information by requiring providers of non-prepacked foods to supply information related to allergen content within their foods.

**Methods:**

Using in-depth interviews with 60 FA/FI adults and 15 parents/carers of FA/FI children, we aimed to identify FA/FI consumers’ preferences for written and/or verbal allergen information when eating out or ordering takeaway food.

**Results:**

A complex and dynamic set of preferences and practices for written and verbal allergen information was identified. Overwhelmingly, written information was favoured in the first instance, but credible personal/verbal communication was highly valued and essential to a good eating out experience. Adequate written information facilitated implicit trust in subsequent verbal information. Where written information was limited, FA/FIs depended on social cues to assess the reliability of verbal information resources, and defaulted to tried and tested allergen avoidance strategies when these were deemed unreliable.

**Conclusion:**

Understanding the subtle negotiations and difficulties encountered by FA/FIs when eating out can serve as a guide for legislators and food providers; by encouraging provision of clear written and verbal allergen information, and training of proactive, allergen-aware staff. This, in tandem with legal requirements for allergen information provision, paves the way for FA/FIs to feel more confident in eating out choices; and to experience improved eating out experiences.

## Introduction

For individuals who experience food allergy (FA) and food intolerance (FI) avoidance of allergens is the key recommended strategy in preventing negative health outcomes. Accidental allergen ingestion is potentially life threatening for many FA individuals [1, 2], and can account for a substantial number of ‘healthy’ days lost in FA/FI populations [3]. Twenty-one to 31% of such accidental allergen ingestion occurs when eating in restaurants and 13–23% occurs in other eating out environments such as work or school canteens [4]. As a result, eating out presents a particular challenge for FA/FI individuals, and is a broader public health concern for legislators, food providers, and the wider community as a whole.

In order to improve the provision of food allergen information for FA/FI consumers when eating out, Europe wide EU legislation was introduced in December 2014. This requires providers of non-prepacked foods to supply written and verbal information related to the content of one or more of 14 specified food allergens within their foods. Within the UK, the Food Standards Agency (FSA) has provided guidance on how allergen information might be provided [5]. However, the guidance regarding the format for delivery of this information is broad and at the discretion of individual eating out providers. Little is known about the preferences for such information provision from FA/FI populations’ perspectives. Understanding these perspectives prior to the legislation’s introduction was vital in order to provide legislators and eating out providers with insights into FA/FI’s information delivery preferences; thereby informing initial and ongoing implementation of improvements in allergen information provision for the benefit of FA/FI consumers.

We explored the allergen-related information delivery preferences of FA/FI populations when eating out or ordering takeaway foods. Results serve to inform legislators in their future recommendations for allergen information provision, and act as a guide of ‘good practice’ for food providers who are required to supply food allergen information for FA/FI consumers.

### Background

Within Europe, FA affects up to 5% of adults and 8% of children [6], and the prevalence of FI is thought to be substantially greater [7, 8]. For FAs, accidental consumption of food allergens accounts for 32.2% of anaphylaxis-related hospital admissions [9], and eating outside the home has been implicated in 50% of deaths related to food allergen consumption [10]. Whilst morbidity and mortality rates are generally low, symptom-based figures underestimate the ongoing impact of food allergen avoidance on FA/FI individuals’ well-being, and decrements in quality of life have been reported alongside significant restrictions in social and behavioural outcomes for these populations [11–14].

The implications of having to exclude one or more foods from the diet can present wide-ranging and unique challenges for FA and FI populations. FA populations describe the need for constant vigilance, with no guarantee that their efforts will be effective in ensuring successful avoidance of the offending food. This has been termed ‘trying to control the uncontrollable’ [15](p. 284). Both FA and FI consumers express concerns regarding the risks posed when consuming foods which they have not prepared; and eating out or ordering takeaway food in particular [16–19]. This apprehension may be justified given literature suggesting a mismatch between restaurant staff’s confidence in their knowledge of food allergens, and the knowledge actually exhibited in practice [20] [21].

EU legislation [22] introduced in December 2014 affects restaurants, takeaway shops, food stalls, institutions like prisons and nursing homes as well as workplace and school canteens. The regulations require food providers to supply customers with accurate and accessible information relating to the inclusion of any of the allergens—peanuts, tree nuts, milk, soya, mustard, lupin, eggs, fish, molluscs, crustaceans, cereals containing gluten, sesame seeds, celery, and sulphur dioxide at levels above 10mg/kg, or 10 mg/litre—in their foods. Allergen information can be provided in written or verbal form. Where verbal information is provided, there must also be written information within the venue that customers can be directed to.

Whilst the intention of the legislation is to provide FA/FI populations with clearer information regarding allergenic ingredients, little is known about how consumers prefer allergen information to be delivered when they eat out—through staff or through written sources of information—or what leads to trust or distrust in these sources. Findings from research into the labelling of pre-packed foods suggest that FA customers combine information seeking strategies by using allergen advice boxes in conjunction with ingredients lists and familiarity cues to minimise their risk of accidental allergen consumption [23]. When offered the option of an information resource in addition to packet labelling, FAs favoured a telephone advice line over an information website; perhaps suggesting that verbal information—though not face to face in this instance—has a particular role in generating trust [24]. The relationship between verbal and written information preferences becomes much more significant when eating out and consuming non-prepacked foods. Although in theory FA/FI individuals have the opportunity to discuss their dietary requirements with staff when eating out, communication difficulties are common; leading to social embarrassment, misunderstanding, and misinformation [16, 17]. This can lead FA/FIs to unduly limit their food selections, or to take unnecessary risks when eating out.

We aimed to understand the preferences and trust cues used by FA/FI individuals when eating out in order to inform the provision of allergen information resources and to outline the implications of this for legislators, food providers, and the wider community. Conducted in the 6 months immediately prior to implementation of EU FIC (1169/2011) legislation, our research is the first to assess the allergen information delivery preferences of both FA and FI populations when eating out; and in particular, their preferences for written and verbal information. This research constitutes phase 1 of the project and ongoing follow-up research will assess the impact of ongoing changes in allergen information provision on FA/FI’s eating out preferences and behaviours.

## Methods

### Recruitment and population

Ethical approval was gained from the University of Bath, Department of Psychology Ethics Committee prior to participant recruitment (Ethical Approval Ref: 14–055). A specialist market research agency recruited 75 participants to complete in-depth interviews. Of the total population, 60 were adults reporting FA/FI, and 15 were parents/carers of children aged up to 17 years with FA/FI. Within the latter group, although the experience of parents/carers was the primary focus of the interview, their FA/FI children were sometimes present and contributed to it. In order to represent the views of consumers throughout the UK, participants were recruited from England, Wales, Scotland, and Northern Ireland. A breakdown of participant characteristics is shown in Table 1.

**Table 1:** Characteristics of the 75 food allergy/intolerance adult participants and children of parent/carer participants.

Variable	Allergy n = 39	Intolerance n = 36	Total (%) N = 75
Sex:			
Male	7 (17.9)	9 (25.0)	16 (21.3)
Female	32 (82.1)	27 (75.0)	59 (78.7)
Age:			
<8	2 (5.1)	2 (5.5)	4 (5.3)
8–12	3 (7.7)	0	3 (4.0)
13–17	4 (10.3)	4 (11.1)	8 (10.7)
18–30	9 (23.1)	8 (22.2)	17 (22.7)
31–45	10 (25.6)	8 (22.2)	18 (24.0)
46–60	5 (12.8)	9 (25.0)	14 (18.7)
60+	6 (15.4)	5 (13.9)	11 (14.7)
Region:			
England	15 (38.5)	17 (47.2)	32 (42.7)
N Ireland	4 (10.3)	6 (16.7)	10 (13.3)
Scotland	10 (25.6)	8 (22.2)	18 (24.0)
Wales	10 (25.6)	5 (13.9)	15 (20.0)

Prior to interview, participants completed a screening questionnaire characterising their or (for parents) their child’s reactions to one or more of the 14 specified allergens. Characteristics were based on nature of reaction, speed of onset, and how FA/FI was diagnosed. This information was used to classify participants as IgE-mediated FA; or non IgE-mediated FA/FI which was either medically or non-medically/self-diagnosed. Thirty-nine participants (52%) were classified as having IgE-mediated FA, and thirty-six (48%) were classified as non IgE-mediated FA/FI. Of the 14 allergens covered by the legislation, FA/FI to peanuts, tree nuts, milk, soya, mustard, lupin, fish, crustaceans, cereals containing gluten, sesame seeds, celery, and/or sulphur dioxide were reported. No participants reported FA/FI to lupin or molluscs.

### Procedure

Following written informed consent, in-depth semi-structured interviews were carried out with participants in their own homes on the basis of an interview protocol detailing questions and possible prompts (a copy of this interview protocol can be provided on request from the corresponding author). Interviews were carried out by RP, JB, or DR, and each interview was audio-recorded with participants’ permission. Initial questions engaged participants with the topic of food and experiences relating to allergy/intolerance diagnoses, adaptation, and day-to-day coping strategies. The interview then focused on participants’ experiences and behaviours when eating out. Participants were encouraged to discuss strategies and environmental/social cues which influenced their decision-making processes; and to consider these preferences in relation to current and future information provision within the new legislation. Interviews lasted between 60–90 minutes.

### Analyses

In order to communicate the diversity of views and perspectives surrounding participants’ eating out experiences, interview recordings were transcribed verbatim and explored in detail using framework analysis [25]. Framework analysis has become popular in social, policy, and health research because it applies a systematic approach to qualitative analysis which prioritises the transparency of the analytical process; thereby maximising accessibility and strengthening confidence in subsequent results and conclusions [26, 27]. Interviews were coded and analysed using QSR NVivo (version 10). Although participants were classified based on their, or (for parents) their child’s, IgE-FA or non IgE-FA/FI status, interviews were analysed across the population as a whole. The analysis was led by FMB and refined and developed in discussion with JB.

Identified themes are illustrated in results. In order to maintain anonymity, participant details are indicated in brackets as follows: A/P refers to Adult/Parent; participant number; country of residence—E = England, S = Scotland, W = Wales and NI = Northern Ireland; and food allergens associated with FA/FI responses. Italicised text reflects interviewer prompts.

## Results

Participants described written food allergen information resources in terms of day to day ‘*use’*, the ‘*adequacy’* of the information, and *‘preferences’* for information provision. Additional theme-based quotes are available in S1 File.

### Use of written information resources

Where possible, participants preferred to rely on written information in preparation for, and during, their eating out experiences. For many, particularly in relation to unfamiliar venues, written information provided the first tangible point of contact on which to base their initial food choices. Preliminary enquiries were made using venue websites to explore food options (Box 1A); and checking recipes of potential meals on the internet (Box 1B). Before committing to dine in a venue, participants gathered information about their potential food options by inspecting menus displayed in the restaurant window (Box 1C). Within the eating out venue itself, participants emphasised the role of the menu in providing detail in relation to ingredients and preparation method (Box 1D and 1E), and additional sources of written information (Box 1F).

**Box 1. Use of written information.**

*Preparation for eating out*:

1. I’ll look usually online—I thank God for the internet—at what their menu is. As I said, before we went to (European restaurant), I’d decided…I’d looked it up online and looked at their menu and gone, right, and I know I had a penne pasta dish….so I knew that one was going to be fine. (A14 G2 S: Milk)
2. If you’re going in a few days, you can Google what the recipe is sort of thing, a rough guide, and you think, mm, that’s okay, and then you just reiterate when you get there, right, I’m allergic to this. So, you know, it’s just basically Googling things… (A39 G1 W: Peanuts, tree nuts, celery)
3. We look in the windows and we try and read, they’ll put a sample menu or whatever, or outside and you try and read what kind of things are in there and if you can see that there is something that you think would be okay then it’s worth a try. (P1 G1 E: Peanuts, tree nuts, milk)

*In the venue*:

- …I’d look at the menu….. I’d sort of look at the list, oh, yeah, I like that one, and then I’d look underneath, which would tell me the ingredients, most times, with most of them, and then I’d order it. (A6 G1 E: Peanuts, tree nuts, cereals containing gluten)
- …it’s fine because it (the menu) normally gives me, 9 times out of 10, it will tell me what’s in the food. So, if I go to a restaurant and there’s a fish, it will tell me how…it will normally say “Cooked with a white wine sauce” or cooked with whatever. It’ll say how it’s served. (A23 G2 NI: Milk)
- Well pizza (chain outlet) ….have the thing on the menu that says if you want to make sure of anything else in the ingredients, take a picture of this QR code, and if you take a picture of the QR code, it takes you to (chain outlet’s) website and you can check yourself. (A33 G1 S: Peanuts, fish)

When written information, on menus in particular, was considered to provide adequate information about ingredients and food preparation, participants reported a sense of autonomy and control when making choices. In part, this normalised the process of their food selections by allowing participants to choose their meals without recourse to additional resources. This in turn gave them greater freedom and a sense of relaxation when eating out.

### Adequacy of written resources

Participants had mixed experiences in relation to the adequacy of written information resources and provided examples of good and poor practice. It was generally perceived that venues which provided more detailed allergen information would be more accommodating and caring towards FA/FI consumers (Box 2A and 2B). For some participants, the experience of poor written resources was variously a source of frustration, annoyance and anxiety; which potentially reduced their enjoyment in the entire eating out experience and caused them to avoid certain venues or eating out as a whole (Box 2C and 2D).

**Box 2. Adequacy of written resources.**

1. I think it was in (chain restaurant)….they’ve just started doing a gluten-free burger….with a gluten-free bun, and they even said…we try our best to avoid cross-contamination… So, when they actually mention that, it’s kind of reassuring that, oh, they actually know what they’re doing. (A13 G2 W: Cereals containing gluten)
2. …if it’s clearly labelled and I don’t have to be the one getting someone to search through a file or go and ask a chef. It makes a massive difference. You just feel comfortable. (A56 G1 E: Egg)
3. Very poor…I think they ought to provide more information. It’s like they brought out that thing with calories now. They put the calories next to the menu, the meal. It’s a good idea but they should do that for allergies as well. A lot of places don’t do that. (P12 G2 W: Peanuts, tree nuts, milk)
4. British restaurants and those sorts of things, they just add wheat to absolutely everything, so it’s impossible…..Things like that really aggravate me, and you find, particularly in restaurants, like the list of ingredients, it’s just not adequate. (A60 G1 E: Peanuts, tree nuts, cereals containing gluten)

### Preferences regarding written information provision

Within the context of the new legislation and more generally, participants had clear, though varied ideas on how best to convey allergen information in a written /visual format. As a basic principle, the overwhelming majority of respondents believed that written information regarding food allergen content in meals should be readily available. Ideally, information provision requiring minimal effort on the part of the consumer, whilst avoiding the potential risk of reliance on staff as intermediaries in information provision, was desired (Box 3A). Expectations regarding the levels of complexity and detail for that information differed however. Many advocated the use of abbreviations or symbols (Box 3B and 3C), or a simple notification inviting further enquiries (Box 3D); whilst others appreciated more detailed allergen information provided as a section within the menu or as a separate and comprehensive written resource (Box 3E and 3F).

**Box 3. Preferences for written information.**

1. If you’re going to be providing information, provide the information—don’t make the customer go and ask for it…Human beings are human and they make mistakes… In a busy restaurant where people are talking to you, you know, you could be given the wrong information actually, so I would like that information provided in written form somehow….I wouldn’t want to have to ask for it. (A52 G1 S: Tree nuts, cereals containing gluten)
2. …they’ve got the “V” and the “N” on the menu, it would need to be a symbol-based thing, I think….. Because if you…had a particular allergy, you would just be scanning the menu for that particular symbol or letter or whatever it may be. I think that would be far more useful than having the huge long list of every ingredient. (A7 G1 E: Peanut, tree nuts)
3. All it’s got to have is a GF next to it and I’m happy. Or even if it says ‘not GF’. It would be better…I think that would be really, really useful, and if it doesn’t do that then I feel like I’m a pain. (P5 G2 E: Cereals containing gluten)
4. …if they just had a nice wee clear “We supply gluten-free” or “Ask our staff”, you know, to provide a list…if you do have any form of intolerances, and we can leave any ingredients out or something. (A30 G3 NI: Cereals containing gluten)
5. …the menu, that “Oh, we’ve got a gluten-free section,”…that is something that they can start doing more, because some people may be embarrassed to talk about it and, you know, not…ask the question. (A4 G2 E: Cereals containing gluten)
6. …they have the list on every single item in there—you know, dressings…sauces…all the allergy ingredients information, is listed on there. So…you know what you’re getting and you know exactly what’s in everything….and they update it as well…so that’s brilliant. (A39 G1 W: Peanuts, tree nuts, celery)
7. I would prefer a simple description, but I have been in restaurants where…I’m not too sure what it means…They maybe list about six different ingredients and…I can recognise so many of them, and some of them, I’m not too sure about. (A20 G1 NI: Peanuts, tree nuts)

Although many participants requested a more detailed menu, it was also recognised that the inclusion of such detail might pose practical problems for menu presentation and readability; particularly in the case of comprehensive ingredient lists within main menus. A minority of participants also raised concerns about their own ability to identify and recognise the relevant allergens listed (Box 3G). Similar reservations in relation to the use of abbreviations/symbols as a more simplified form of allergen warning were also highlighted. Although this was a preferred method of information delivery for many, a small number of respondents raised questions relating to the consistent use of symbols across venues and countries, and the potential for confusion and accidental allergen ingestion that might result from the inconsistent application of symbols or abbreviated messages.

### Verbal information resources

As an inherently social experience, participants reported that the seeking of verbal information relating to food allergens within dishes varied based on their familiarity with the eating out venue. In regularly attended venues, where a successful track record of eating out had been established over time, participants valued the feelings of confidence and relaxation which resulted from their previous interactions with helpful and accommodating staff. In unfamiliar venues, where no such prior relationships had been established and written information was judged to be incomplete, participants used a number of cues to assess the reliability of the allergen information provided by staff. Primarily, participants based these assessments on staff knowledge and more subtle perceptions of staff interest, engagement and attitude with regard to their dietary needs. Where staff knowledge (Box 4A and 4B) and demeanour (Box 4C and 4D) were deemed to be good, trust and confidence in the safety of their meal was raised. Equally, the opposite was the case when knowledge (Box 4E and 4F) and demeanour (Box 4G and 4H) was deemed to be poor.

**Box 4. Staff knowledge and demeanour.**

1. The (Asian restaurant), as I said, they done gluten-free. They were able to offer an alternative to soy sauce and everything. So, she was able to say, ‘Well, you can’t have noodles but you can have rice noodles.’ So, she was actually more knowledgeable than me on coeliac, so that was good. (A48 G2 S: Cereals containing gluten)
2. (Sandwich chain) are usually quite good because I…went to one a couple of years ago now, and I said, “Oh can I have that, but I’m allergic to cucumbers so you’re going to have to completely…” you know, and she said, “Well, that’s cut in the same machine, so you can’t have that.” So, they kind of know…what’s cut what and what’s doing what. So, (Sandwich chain) are quite good for knowing what’s in the products and stuff. (A39 G1 W: Peanuts, tree nuts, celery)
3. You get some people that are quite perky and cheery and…Also, asking specifically as well… So, I’d say, like I might accidently say “No milk” and they’d be like “Do you also not want cheese?” or “No prawns” and they’re like, “Are you okay with..?” you know, this other thing. So, you know, you get some people that are quite on the ball in that sense. (A11 G1 S: Milk, Crustaceans)
4. …if a waiter is really keen on like listening and just writing all the ingredients, just to make sure she speaks or he speaks to the chef. So, yeah, just basically communication and the way they treat those things. (A9 G1 E)
5. The trust is in the staff, to begin with. I mean, they’re your first contact, aren’t they? If they have knowledge of the food, then I’m quite confident. If they have no knowledge of the food, then I think I’m not coming here again. (A18 G3 E: Milk)
6. Some do say, “What do you mean, dairy, what do you mean?” and I say cream, cheese, milk, anything like that, and…what makes me laugh, people think I’m going to be allergic to mayonnaise because it’s from the eggs, and…I said, “Actually, it’s not dairy, even though it’s from the hen, it’s not dairy, it’s not a cow… (A26 G2 E: Milk)
7. There’s been times in the past when I know…I can read people, and I know that they’re thinking “Oh, for God’s sake, this is a fad!” sort of thing, you know, and it’s not good enough. (A18 G3 E: Milk)
8. I’ve had them just shrug their shoulders and say “I don’t know.” “Well, does the chef know?” “I don’t think he will,” you know, sort of thing…and you’re thinking, you’re joking…! (A53 G2 E: Cereals containing gluten)

Participants identified other factors which inspired trust or served as barriers to their perceptions of staff members as reliable information resources. Younger staff members were viewed as inherently less reliable as information resources. This was largely due to an absence of life experience, and the potential for a lack of personal investment in their appointed roles. For some, this perceived lack of reliability did not necessarily lie with young frontline staff per se, but pointed instead to a potential systemic problem relating to eating out establishments as a whole. Better training was thought to hold the key to greater levels of trust and confidence in the information provided by staff.

Whilst a minority of participants sought verbal information as a safety clarification in addition to written information resources, the majority reported a sense of reluctance and embarrassment when making enquiries of staff. Although asking questions of staff was seen as a necessity by many participants; for others the perceived embarrassment of asking staff for further information led to self-imposed limitations in food selections, or unnecessary risk taking.

## Discussion

Written information of sufficient quality was used as a baseline resource which liberated FA/FIs to make their food selections independently and without recourse to other information seeking strategies. Beyond the written resource itself, FA/FIs inferred a wider message of ‘understanding’ on the part of venues that provided adequate written allergen information, and were reassured by notices encouraging customers to ask staff about the allergen content of foods. This implied awareness gave FA/FIs permission to ask questions of staff with the expectation of an informed response; and without fear of embarrassment. At its best, accurate and trustworthy food allergen information delivered verbally by staff also enhanced FA/FI’s eating out experience. Judgements regarding the potential for accidental exposure to food allergens were contingent on subtle social cues suggestive of staff knowledge; and were assessed by FA/FIs accordingly. Where doubts surrounding verbal allergen information occurred, FA/FIs retreated to their default position of reliance on written information resources, and in turn limited the potential variety of venues and food options available to them as a result. However, with adequate written allergen information, and the positive interactions of reliable allergen-aware staff; FA/FIs experienced an increase in trust and loyalty to eating out/takeaway venues concerned.

Fundamental to FA/FI’s concerns surrounding allergen information provision when eating out, was the need for constant vigilance to ensure allergen avoidance, balanced against a wish to avoid ‘drawing attention’ [16]. EU FIC (1169/2011) legislation has the potential to address these issues by making the provision of food allergen information mandatory, thereby validating and normalising food allergies and intolerances. By empowering FA/FIs with the right to ask and expect adequate information provision, it is to be hoped that the latter fear of embarrassment and resultant social isolation will be reduced [14, 17].

Given that strict allergen avoidance is necessary for many FA/FIs [28, 18] and the risk of food allergen exposure when eating out is high [4], our research indicates that FA/FIs clearly have no coherent set of preferences for the delivery of allergen information within an eating out setting. At its best, legislators should aim to cater for this diversity of preferences by recommending a combination of written and face to face allergen information provision to accommodate the varying needs and preferences of FA/FI populations. Food providers can play a crucial role in meeting FA/FI’s needs through the provision of clear written allergen information, increased allergen-awareness training for staff, and effective communication mechanisms between food preparation and serving areas. Alongside written information, our results indicate that staff use of simple, proactive face to face strategies to make enquiries and reassure customers, is favoured by FA/FIs. For example, training staff to ask diners about any food sensitivities from the outset, would convey allergen awareness, and would likely diminish much of reticence exhibited by FA/FIs within this study and in wider literature [14, 16].

In recognising the insights gained through the in-depth analysis of FA/FIs information preferences when eating out, we also acknowledge the limitations of the study. Given that we were seeking to understand the perspectives of those with both FA and FI it was necessary to use self-report measures to assess FA/FI status. Although this was done through the careful application of strict symptom-based FA/FI criteria; the assignment of some participants presented a challenge. However accuracy of allocation was less critical within the remit of the current study which sought a broader perspective on FA/FI populations’ preferences for written and/or verbal food allergen information when eating out. Due to the qualitative nature of our research we were also unable to account for the impact of demographic factors such as sex, age and region of residence within the UK. These factors may have affected FA/FI’s preferences in terms of allergen information provision and willingness to communicate with staff.

### Conclusion

In light of EU legislation requiring that eating out providers supply consumers with information regarding the allergen content of their foods, this study is the first to gain in-depth insights into FA/FI consumers’ preferences for the provision of allergen information when eating out or ordering takeaway foods. Findings indicate that FA/FI consumers were often ambivalent or conflicted in their preferences for written and verbal allergen information provision. FA/FIs overwhelmingly favoured tangible, written information in the first instance; and adequate written information often led to an implicit trust in subsequent verbal information. Where written information was limited, FA/FIs depended on social cues to assess the reliability of verbal information resources, and defaulted to tried and tested allergen avoidance strategies when these were deemed unreliable. Understanding the subtle negotiations and difficulties encountered by FA/FIs when eating out can serve as a guide for legislators and food providers; by encouraging the provision of clear written and verbal allergen information, and the training of proactive, allergen aware staff. This, in tandem with legally enforceable requirements for food allergen information provision provided by the EU legislation, paves the way for FA/FIs to feel more confident in their eating out choices; and to experience a safer eating out experience.

## Supporting Information

S1 File Additional theme-based quotes from interviews. (DOCX)
