# Supplementary material for: Consumer Preferences for Written and Oral Information about Allergens When Eating Out
Source: PLoS One. 2016 May 25;11(5):e0156073. doi: 10.1371/journal.pone.0156073 (PMC4880205; doi:10.1371/journal.pone.0156073)
Supplement: S1 File — (DOCX) [file pone.0156073.s001.docx]

**S1 File: Additional theme-based quotes from interviews**

**Use of written information**

[<Internals\\A1 G3 E>](file:///F:\Food%20allergy%20&%20intolerance\Papers\Paper%201%20submission\PLOSone\PLOSONE%20resubmission\e41d632e-c0b0-4f4b-88d1-7a2af64b72b3) -

Again, the descriptions are…sometimes, they’re okay. Other times, they’re very vague, and it’s those ones that then I think, oh, what am I going to do now?! You know, I don’t know whether to phone them and say, you know, before I go, because then I’ve spoken to them, they don’t know who I am because I don’t give my name, and then, when I go, they won’t say “Oh, you were the guy that phoned up…”! Yeah, so, yeah, I try and…I try and do that if it’s not as informative as it could be, and very often they’re not, they’re not at all. They don’t say, em…sometimes they do, but very often not “with a creamy sauce” – it just says “served with chips” and that’s no good to me, you know. Yeah.

[<Internals\\A11 G1 S>](file:///F:\Food%20allergy%20&%20intolerance\Papers\Paper%201%20submission\PLOSone\PLOSONE%20resubmission\34e7524f-e359-45ca-99d1-780a919588c7)

I think, by default, I know that I can eat in most places, apart from maybe Italian, and I’d be guaranteed to find something that I can eat. So, I think I would mostly just check menus if a friend had recommended it and said “Do you want to come to this place?” and I wanted to check the price, and maybe, you know, if they hadn’t described it, maybe check if, you know, if it wasn’t Italian – that really is my cut-off point. Or a shellfish restaurant!

[<Internals\\A12 G3 S>](file:///F:\Food%20allergy%20&%20intolerance\Papers\Paper%201%20submission\PLOSone\PLOSONE%20resubmission\b7ba2429-fb15-41e1-afd1-780aa00fc7ed)

I mean, it’s getting bad up here in (place name) when you eat out, but so far, I’ve managed to get away with it, but I find now, when I’m going down South, it’s getting harder, and if we go out eating with my son and his wife, and maybe go to places, like we were at (place name) the other…in (month), and we ate down there, and that was okay. Then we went to somewhere else which wasn’t – it had spicy things in it….

[<Internals\\A13 G2 W>](file:///F:\Food%20allergy%20&%20intolerance\Papers\Paper%201%20submission\PLOSone\PLOSONE%20resubmission\b02abc14-81eb-47b9-a7d1-780aaff3d798)

Yeah, I’d go to the menu, and if I can’t see anything that specifically says gluten-free or GF or like separates it from the rest, I’d just eat something that I know for a fact is gluten-free just because it is, like a natural food like a potato or fish or…

[<Internals\\A16 G1 E>](file:///F:\Food%20allergy%20&%20intolerance\Papers\Paper%201%20submission\PLOSone\PLOSONE%20resubmission\83ee4d86-4c0c-4e9b-b0d1-780b2b46c1fd)

Well, I think the first thing…I think the first point of contact is look at the menu. You know, if you sort of look at it and you think, right, it’s chicken korma, so you think, right, well, I know what goes into a korma, and it’s chicken.

[<Internals\\A17 G3 E>](file:///F:\Food%20allergy%20&%20intolerance\Papers\Paper%201%20submission\PLOSone\PLOSONE%20resubmission\03cf7684-d588-4a8b-bdd1-780b43f1e4fa)

Em, mainly the menu and then ask. Sometimes it’s not much use asking the waitress or a waiter, if they’re a young person, but if you can get somebody that’s sort of near the chef or…you can ask them.

[<Internals\\A18 G3 E>](file:///F:\Food%20allergy%20&%20intolerance\Papers\Paper%201%20submission\PLOSone\PLOSONE%20resubmission\51c6d19e-de12-49e2-9fd1-780b598de215)

I’d look at the menu, and then…and then I’ll look at the board to see what’s on, and then, if there’s something that I prefer from the board, I would ask the staff what’s in that….Oh, definitely, definitely look at the menu, yeah, yeah. If it says with a sauce or a marinade, then no, no. Even if it…if it’s a specific dish that comes with a sauce or a marinade, I usually say “Can you please leave off the marinade or the sauce?”

[<Internals\\A19 G2 NI>](file:///F:\Food%20allergy%20&%20intolerance\Papers\Paper%201%20submission\PLOSone\PLOSONE%20resubmission\e9119547-10cd-43a4-81d1-780b6d6a5dab)

Em, yeah, sometimes, places that I know have gluten-free, that I’ve looked up or researched or have had to make a phone call or email them before I would even go. I wouldn’t really…I wouldn’t very often do it spontaneously when I’m out and just decide to go out or to new places that I don’t know if they have it or not, because I find that annoying, going round all the different places and finding out that they don’t have very much… I had to just do my research in advance, so I just looked up on the internet before we went out anywhere and we just…we found that place, so they had gluten-free options. So, I went there and they had…they had very good options and they understood and everything, so they were able to give me, you know…

[<Internals\\A22 G1 NI>](file:///F:\Food%20allergy%20&%20intolerance\Papers\Paper%201%20submission\PLOSone\PLOSONE%20resubmission\4e29355b-621c-4782-bed1-780ba675f744)

Check on the internet, look on my smartphone and get them to point me through to something that looked suitable.

[<Internals\\A23 G2 NI>](file:///F:\Food%20allergy%20&%20intolerance\Papers\Paper%201%20submission\PLOSone\PLOSONE%20resubmission\d68a40bb-95a9-409b-96d1-780bbb80c1eb)

If we were planning to go for dinner, yeah, we’d have a look. We’d look online and see where there is in the area for dinner, and kind of that thing, because you’d ring and book. So, we would probably more than likely do that. If we were in the town down there, let’s say you went [down to the south] even and you’re going shopping or whatever, just probably you just go look round town and see what’s in the town or what kind of looks right or what kind of menus look like they’re….Just eh…if it was somewhere I didn’t know, I would just go online and kind of be like what area are we in…, what’s around this area? And…you get, like (website name), they have restaurant advisor meals. If I was in that situation, that’s what I’d do. I’d go by general consensus of 8 out of 10 people think this place is okay – right, we’ll got there, I’ll get it from there.

[<Internals\\A24 G1 NI>](file:///F:\Food%20allergy%20&%20intolerance\Papers\Paper%201%20submission\PLOSone\PLOSONE%20resubmission\ac115d93-00c6-47cc-b8d1-780beaf8d184) -

First of all, it would be by the style of restaurant, I’d feel, if there’s….if they serve the types of food that I would usually eat, I would go there, and then I would ask them, as soon as you go in then, if they’re… Usually, if they’re not happy, they’ll tell you [laughing]. They won’t be slow in saying.

Look at the menu, yeah. Sometimes, if it’s a new restaurant, I’d phone them, you know. If I’m going with a group, I would generally phone them and see rather than having to go the whole way there and get told you can’t…there’s nothing suitable.

Usually look at the menu and look at something that you would feel most…most confident with. Quite often, I would ask for, generally, without the salad because, quite often, it comes with coleslaw, which is mayonnaise in it or salad cream, and if that touches, you know, sometimes that can be enough. So, quite often, I just get quite plain, plain dishes, and I’d ask, you know, generally I’d ask about any sauces that I wasn’t familiar with.

[<Internals\\A27 G1 W>](file:///F:\Food%20allergy%20&%20intolerance\Papers\Paper%201%20submission\PLOSone\PLOSONE%20resubmission\0264b4c7-aa60-43a7-aed1-780c25a4fc11)

Well, when you go into a restaurant or a café, is there a notice there that says, you know, we do use peanuts, something along that line, or something to say, well, “If you’ve got a peanut allergy, please ask questions”, which you never, ever see anywhere.

[<Internals\\A28 G1 W>](file:///F:\Food%20allergy%20&%20intolerance\Papers\Paper%201%20submission\PLOSone\PLOSONE%20resubmission\df9f8d1a-9ed7-4cf7-96d1-780c3af72ff1)

Well, if we go somewhere, we always tell in advance, when we’re booking, we’ve got a member…well, me, member of the family that’s… People are really, generally, very good.

[<Internals\\A29 G3 S>](file:///F:\Food%20allergy%20&%20intolerance\Papers\Paper%201%20submission\PLOSone\PLOSONE%20resubmission\10dca81f-5134-4a0f-abd1-780c506f78da)

So, yeah, I will definitely check menus and stuff online, and especially going anywhere with my (parents), I would be quite scrupulous about that because I feel my (parent is) more sensitive….Places normally have a menu in the window, to check before you go in so that you’re not looking like an eejit.

So, but again, the pre-checking thing, I’m never going to just drop in to a high-end restaurant. I’m not moving in those circles where that would be an option. So, if I’m going somewhere that would be considered high-end, it will have been a pre-considered, researched, menus checked online…

[<Internals\\A3 G3 E>](file:///F:\Food%20allergy%20&%20intolerance\Papers\Paper%201%20submission\PLOSone\PLOSONE%20resubmission\284cfbd0-b520-4716-80d1-7809ec89df6c)

If I could go in somewhere – first of all, if I went in somewhere and it said… If the menu is on the wall and said up there that it was sort of dairy-free or, you know, then you wouldn’t mind asking somebody, but if it’s not then…

[<Internals\\A31 G1 S>](file:///F:\Food%20allergy%20&%20intolerance\Papers\Paper%201%20submission\PLOSone\PLOSONE%20resubmission\78b2500b-e00b-46f7-bcd1-780c7b7a3a64)

No, I mean, we would just, like anybody else, just look at the menu, either online or in the window, and then look at the prices, decide whether we were going to go, and the nut allergy conversation would happen inside.

[<Internals\\A32 G2 S>](file:///F:\Food%20allergy%20&%20intolerance\Papers\Paper%201%20submission\PLOSone\PLOSONE%20resubmission\1310ff29-9ef0-44b1-a1d1-780c8f995c68)

If we were somewhere else in the UK, I would probably, em, I would probably stick to the kind of, you know, nationwide chains that I know are going to have a different variety, just to be sure maybe. You know, if there’s not, I’ll just…I’ll risk it and just have something boring

I think there’s a lot of information about, especially because, you know, you can look it up. You can look at these things online, and I get a lot of things sent to me as well from Coeliac UK, so I feel like there’s a lot of information for me, and as well for other people that have, you know, kind of similar food allergies.

[<Internals\\A35 G1 W>](file:///F:\Food%20allergy%20&%20intolerance\Papers\Paper%201%20submission\PLOSone\PLOSONE%20resubmission\12135680-7bc1-4575-add1-780cd710ecf3)

Em…well, you’d have a look at the menu outside first and see if I fancy anything there, and that that hasn’t got any allergy affecting me, I’d go in.

[<Internals\\A38 G3 W>](file:///F:\Food%20allergy%20&%20intolerance\Papers\Paper%201%20submission\PLOSone\PLOSONE%20resubmission\909439ae-b553-41c3-9fd1-780ef23fbe1f)

I’ll look at the menu, and I’ll have, you know, I’ll have a look, and then, like I say, if I’m not sure, then I will ask. Or I’ll stick to something that I know, you know, that I think…because…I don’t know, because, you know, it is sort of…you do feel that, if you’re asking things, that you’re being sort of a bit awkward.

[<Internals\\A42 G3 W>](file:///F:\Food%20allergy%20&%20intolerance\Papers\Paper%201%20submission\PLOSone\PLOSONE%20resubmission\a4942d1f-401f-4da0-97d1-780d5cc6b8f3)

Yeah, you’d have to totally scrutinise the menu to try and… And again, yeah, you end up asking as well, you do end up asking them, because sometimes they don’t…you know, I can imagine they don’t put all the ingredients, so you do have to end up checking, which isn’t ideal.

[<Internals\\A43 G1 S>](file:///F:\Food%20allergy%20&%20intolerance\Papers\Paper%201%20submission\PLOSone\PLOSONE%20resubmission\b78f1290-ec1b-4e9b-90d1-780d7006196d)

I’m a menu-looker, yeah. Unless…unless I’m particularly… I’m thinking about (city), unless I’m particularly drawn into somewhere that just looks good… You can usually in (city) because, if there’s lots of people, it’s good, and if there isn’t and there’s… My rule of thumb is, if someone’s serving food and they’ve got pictures of the food in laminate, walk the other way.

That’s usually my rule of thumb. I’m a menu-reader though. If something grabs me on the menu… Being vegetarian as well, I’m usually limited for choice. If I see something on the menu, that would usually pull me in the door more than the sort of ambience look of the place.

[<Internals\\A44 G1 E (G2 instead)>](file:///F:\Food%20allergy%20&%20intolerance\Papers\Paper%201%20submission\PLOSone\PLOSONE%20resubmission\f74714b6-db56-491b-bed1-780d8cb32ccf)

A lot of the times, they have a menu outside now, these places, so you can kind of view it before you go in,

[<Internals\\A46 G2 E>](file:///F:\Food%20allergy%20&%20intolerance\Papers\Paper%201%20submission\PLOSone\PLOSONE%20resubmission\c84dee5c-69a7-491e-a0d1-780db538d920)

I’m going to try, yeah, I’m going to try and see if they’ve got a website. It seems to be like an independent. I don’t think it’s part of a chain. But I’ll look on their website and see if they’ve got one and see if I can have a look at the menu. I’ve not eaten in there before. So, em, that tends to be what I do if I know where I’m going.

[<Internals\\A39 G1 W>](file:///F:\Food%20allergy%20&%20intolerance\Papers\Paper%201%20submission\PLOSone\PLOSONE%20resubmission\7f01cf85-a7f7-47a8-9bd1-780d2352613c)

Yeah, yeah, just look where you’re going – ooh, that looks nice [laughing], kind of plan what we’re having to eat before you even get there!

[<Internals\\P2 G1 E>](file:///F:\Food%20allergy%20&%20intolerance\Papers\Paper%201%20submission\PLOSone\PLOSONE%20resubmission\9fde6335-bd8c-4ecb-a0d1-780fa28280aa)

Enough for me to decide whether its somewhere we could go. I’d probably look into it a little bit more. If he said can we go to a (restaurant type) I’d probably say ok, I’d already have an idea of whether its somewhere we could go. And then if it did come up, then I’d probably make a bit more of an informed decision. Go and have a look to find out a little bit more.

[<Internals\\A30 G3 NI>](file:///F:\Food%20allergy%20&%20intolerance\Papers\Paper%201%20submission\PLOSone\PLOSONE%20resubmission\c45cc090-bb90-40d5-b5d1-780c6500dc6d)

Oh, (search engine), obviously, and have a look, and hopefully word of mouth, you know, but I think there is quite a good wee directory going on sort of on that system, you know, for that (self-help orgnaisation) anyway. You can actually put into them, you know, and they’ll give you a list of different restaurants and things in the local area.

[<Internals\\A25 G1 E>](file:///F:\Food%20allergy%20&%20intolerance\Papers\Paper%201%20submission\PLOSone\PLOSONE%20resubmission\36cc6cd9-6f03-4df2-94d1-780bfedc702d)

Em, probably the website. I’d ask the staff, usually the manager, just to be sure. And the menu I suppose, yeah…

[<Internals\\A35 G1 W>](file:///F:\Food%20allergy%20&%20intolerance\Papers\Paper%201%20submission\PLOSone\PLOSONE%20resubmission\12135680-7bc1-4575-add1-780cd710ecf3)

No, it’s just have a look at the menu and then, em, usually, the menus, they say what’s in the menu, and then I’m quite happy. If I’m not, well, I will ask the staff.

[<Internals\\A40 G1 W>](file:///F:\Food%20allergy%20&%20intolerance\Papers\Paper%201%20submission\PLOSone\PLOSONE%20resubmission\922ffa83-21ec-43ad-bbd1-780d35f24675)

Have a look at the menu and then go in and ask them, because if there is something that I like on the menu, then they might be able to cook it without nuts or whatever in, so yeah.

[<Internals\\P6 G1 S>](file:///F:\Food%20allergy%20&%20intolerance\Papers\Paper%201%20submission\PLOSone\PLOSONE%20resubmission\c86011b3-5347-424b-84d1-781079ecb721)

I’m a big Internet lover, I like to check things out. I do look at menus beforehand, some menus will say if it’s got something in it, others won’t. But you’re always going to take a gamble. I think we do play safe.

**Written information preferences**

**Signs & symbols**

[<Internals\\A10 G3 S>](file:///F:\Food%20allergy%20&%20intolerance\Papers\Paper%201%20submission\PLOSone\PLOSONE%20resubmission\fcd555ac-59c4-4534-86d1-7a2b4b943fc1)

I think they produce their own things, so it shouldn’t be too hard. They’ll just have to sort of bring in a system in place that, whoever it is they get it from – I mean, I think it’s done specially for them – will just have to tell them what’s in it. And as for producing a little sign, I don’t think that will be too hard, and there’ll be a slight, you know, extra expense, but I don’t think it’s going to be that hard for them…. I mean, they already have the little V symbols for vegetarian, so I imagine they’d just put something in like that, and they might, you know, put a little “C” for coeliac-friendly or something. Yeah.

[<Internals\\A13 G2 W>](file:///F:\Food%20allergy%20&%20intolerance\Papers\Paper%201%20submission\PLOSone\PLOSONE%20resubmission\b02abc14-81eb-47b9-a7d1-780aaff3d798)

.the second I see like a key, like along with the V, vegetarian sign, if there’s like there NGCI, so no gluten in any ingredients, if I see that, or if I see GF for gluten-free, and if I can clearly see that key at the bottom, and then it related to certain meals on the menu, that’s when I feel completely fine, like they’ve taken the time to make it apparent what I can eat, or, even better, if there’s a separate gluten-free menu.

[<Internals\\A15 G1 E>](file:///F:\Food%20allergy%20&%20intolerance\Papers\Paper%201%20submission\PLOSone\PLOSONE%20resubmission\96e8102c-6039-4058-84d1-780b13e67e6a)

I can use the internet now and find other people who have these allergies, and say why don’t we have an icon where, immediately, you can see if one of these is in there, because there’s a picture of something on the front of the package, so I can immediately see that it’s got one of those things. It might be one of several different colours or there might be several different icons, the same way as if you, you know, if you buy a film or a game, if it’s not suitable for kids, it will have a 15 or 18 on it, and then you know, that that’s got content that’s not suitable for children. I want to immediately, on the front, front and…not necessarily centre, but see an icon immediately, and I know that, okay, I need to be aware.

[<Internals\\A19 G2 NI>](file:///F:\Food%20allergy%20&%20intolerance\Papers\Paper%201%20submission\PLOSone\PLOSONE%20resubmission\e9119547-10cd-43a4-81d1-780b6d6a5dab)

A lot of the menus could have, you know, wee gluten-free symbols beside things, you know, to help people like that, but they don’t so it’s quite hard really….So I think that would be helpful. Because (place name), on their menu, it has a wee “G” or a “V” beside things and then I know that that’s gluten-free, so then it’s easier for me. And whenever they label that, it’s easier, on the menu. It just makes it nicer for me.

[<Internals\\A22 G1 NI>](file:///F:\Food%20allergy%20&%20intolerance\Papers\Paper%201%20submission\PLOSone\PLOSONE%20resubmission\4e29355b-621c-4782-bed1-780ba675f744)

Certainly, it should…there should be warning, some sort of standard warning that you can just very, very quickly look at whatever it is, whether it be a packet of food in the supermarket or on the menu as you go into a restaurant, if there’s something, a warning there that is general to everyone, and as soon as you look at that, you can recognise it. I don’t think we’re in…we’ve got up to that level where it’s easy to see those warnings, so how you can improve them, who knows, but there must be better ways of giving people the information.

[<Internals\\A23 G2 NI>](file:///F:\Food%20allergy%20&%20intolerance\Papers\Paper%201%20submission\PLOSone\PLOSONE%20resubmission\d68a40bb-95a9-409b-96d1-780bbb80c1eb)

I’m full-on with that, but I don’t see why, if we’re doing a menu, like say that’s your two lines containing thing, why down here, with a star beside it, can’t you just put down in green writing, which everybody knows means it’s in the food, nuts, peanuts, fish, eggs, celery, so people know we’re looking at, okay, that already has…

[<Internals\\A24 G1 NI>](file:///F:\Food%20allergy%20&%20intolerance\Papers\Paper%201%20submission\PLOSone\PLOSONE%20resubmission\ac115d93-00c6-47cc-b8d1-780beaf8d184)

Some of them would just say, you know, “Some of our products may contain nuts”, but then, em, quite often, especially in the last few years I’ve found, you know, beside each individual dish, they’d have an N for nuts or… I find nuts, more than eggs, the labelling is better for nuts.

[<Internals\\A29 G3 S>](file:///F:\Food%20allergy%20&%20intolerance\Papers\Paper%201%20submission\PLOSone\PLOSONE%20resubmission\10dca81f-5134-4a0f-abd1-780c506f78da)

Well, one of the kind of helpful things is when they do the wee symbols, you know, for each option, like the wee “I” or whatever…If they do that, it just immediately alerts you, and even if it’s just a wee bit down at the bottom says, “V” mean, and this means, and that means…Yeah. That’s what I would expect. And it wouldn’t be difficult to make up a code for that and include that.

[<Internals\\A30 G3 NI>](file:///F:\Food%20allergy%20&%20intolerance\Papers\Paper%201%20submission\PLOSone\PLOSONE%20resubmission\c45cc090-bb90-40d5-b5d1-780c6500dc6d)

Oh, something actually on there, even a symbol or something to say that, you know, if you do have a gluten or whatever problem, just speak to us, you know, to sort of like arrange for that to be…because, again, you know, if you’ve [good, straight] foods and products and stuff, you can’t mess with it, do you know, and you can actually, if you don’t have a milk allergy or something, have quite a few, you know, ingredients that are in things, but if it’s actually saying we are perhaps friendly and sympathetic to this, you know….I don’t want to be putting…bothering people, I just want to see. I want it to be bold and I want it to be frank, you know, and there’s just like a wee cross that just says it, you know, wheat, you know. There is a symbol. There’s symbols on everything for it, and that’s what I would like to see down the side of the menu because then you’d go, okay, number 32, you know. I don’t know, yeah, yeah…

[<Internals\\A34 G2 W>](file:///F:\Food%20allergy%20&%20intolerance\Papers\Paper%201%20submission\PLOSone\PLOSONE%20resubmission\e6390bbf-351c-4055-a2d1-780cc4ef315d)

I find they’re quite peanut-aware now, but it seems to be like, em, nut allergies have taken over, and it’s just a nut allergy. I actually don’t like nuts anyway, but that’s the only thing…. You think, oh, like peanut allergy or…that’s it, really. And although you do get lots of signs on menus, it takes you an age to work out what the blinking signs are.

[<Internals\\A44 G1 E (G2 instead)>](file:///F:\Food%20allergy%20&%20intolerance\Papers\Paper%201%20submission\PLOSone\PLOSONE%20resubmission\f74714b6-db56-491b-bed1-780d8cb32ccf)

You know how you see like the little V saying it’s vegan or vegetarian, whichever one they decide to use it for, the same – they could a sign, just if it’s got this sign, it means it will contain dairy. Because dairy, if you want to generalise, and if it was your choice or not, if you wanted to be a vegan, or if you were like me and won’t eat it because of your health, you know, that is still the same answer, isn’t it?

[<Internals\\A46 G2 E>](file:///F:\Food%20allergy%20&%20intolerance\Papers\Paper%201%20submission\PLOSone\PLOSONE%20resubmission\c84dee5c-69a7-491e-a0d1-780db538d920)

Like, some of the menus will have gluten-free, but if there was, em - you know, if you look at a menu and it has a big green V, you know it’s vegetarian. That’s kind of a, you know, used across the board. I’ve seen things with a little circle with a wheat, bit of wheat, with a cross in it, em, somewhere, and then somewhere else where they just write GF next to it. So, if there was like a nationally-recognised symbol that you could put, that would be okay. You know, some places, the information is good; some, some not, but I think, as a generalisation, not very good really.

[<Internals\\A48 G2 S>](file:///F:\Food%20allergy%20&%20intolerance\Papers\Paper%201%20submission\PLOSone\PLOSONE%20resubmission\92df34fb-0285-4959-83d1-780ddaefa70d)

No, I never trust symbols, ever, no matter what restaurant you’re in. I just never trust it. The food might be gluten-free, but again, it could be contaminated.

[<Internals\\A49 G1 E>](file:///F:\Food%20allergy%20&%20intolerance\Papers\Paper%201%20submission\PLOSone\PLOSONE%20resubmission\045461f6-fb51-4d35-bbd1-780dec8c15be)

Em, yeah, I think probably, like if you were…especially, you know, on the front of the menu, because you tend to order takeaways from a menu, that you’ve got a kind of paper one, and I think, if they had like symbols and signs or like some sort of…that would definitely, you know… I’d be able to try different types of sushi to what I always go for and, em, yeah, I think, with takeaway menus, that would be really helpful.

[<Internals\\A53 G2 E>](file:///F:\Food%20allergy%20&%20intolerance\Papers\Paper%201%20submission\PLOSone\PLOSONE%20resubmission\8f3a67a9-c6c3-4bb5-acd1-780e420ba2f8)

But there’s very few restaurants put “gluten-free” on. I think (pub chain) is the only one that I’ve come across that actually actively put “gluten-free” on. It’s in like a little bar, green bar, and it’ll say “gluten-free”.

You can see it, you know. If it’s got something the size of this, you know, “gluten-free”, just a little label, “gluten-free”, people will learn where to look for it, and they catch on, and it’s unobtrusive, and you can make a decision without anybody ever knowing that you’re gluten-intolerant.

[<Internals\\A57 G2 E>](file:///F:\Food%20allergy%20&%20intolerance\Papers\Paper%201%20submission\PLOSone\PLOSONE%20resubmission\2cfe0d8b-6bd8-498d-88d1-780ea256cc44)

And you’ve got your little coeliac sign, so I don’t really know why that can’t be put against things, so somebody who is gluten-free would think, right, I can…I don’t need to ask in front of everybody, I can just go down it…

[<Internals\\A58 G1 E>](file:///F:\Food%20allergy%20&%20intolerance\Papers\Paper%201%20submission\PLOSone\PLOSONE%20resubmission\e23e35d0-1b9b-4713-98d1-780eb4ace6d9)

On all menus, it should just say what has nuts in and what doesn’t have nuts in….So, for example, if it’s got gluten in, for a coeliac person, then it says “G”, like for a vegetarian has a “V”, and for a nut allergy, it should have an “N”, something like that, something very straightforward which people understand.

[<Internals\\A59 G1 S>](file:///F:\Food%20allergy%20&%20intolerance\Papers\Paper%201%20submission\PLOSone\PLOSONE%20resubmission\d8a0face-1668-42ec-8ed1-780ec9326302)

Em, I guess it would be helpful if they just had a little sign next to items on the menu that have nuts in, but, I don’t know, I manage. It’s fine.

[<Internals\\A6 G1 E>](file:///F:\Food%20allergy%20&%20intolerance\Papers\Paper%201%20submission\PLOSone\PLOSONE%20resubmission\37f504f9-f51f-4f42-93d1-780a2e934c5a)

I think pubs do that a bit more, if I’m thinking right. (pub chain) have got that on there. Yeah, em, could probably be more visible, but then so could the “V” I suppose, for vegan – you know, how big do they make these things for people? But of course, I mean, it’s great, I think, if they’re telling you, but I don’t want it to tell me that it may have nuts – I just want nuts or no nuts, do you know…I think they do quite well. Like I said, they’ve got it on the menus. If there’s nuts in ingredients, it’s listed. Some of them have the symbols, and you’ve also got the…at the counters, you’ve got the labels that tell you. They could probably do a little bit more in terms of the boards and things like that – could they not put the “N” next to it and stuff there when they’re writing it up?

[<Internals\\A7 G1 E>](file:///F:\Food%20allergy%20&%20intolerance\Papers\Paper%201%20submission\PLOSone\PLOSONE%20resubmission\ef88cc58-116d-40e7-8ad1-780a4402104e)

because if you were…had a particular allergy, you would just be scanning the menu for that particular symbol or letter or whatever it may be. I think that would be far more useful than having the huge long list of every ingredient.

[<Internals\\P12 G1 W(51)>](file:///F:\Food%20allergy%20&%20intolerance\Papers\Paper%201%20submission\PLOSone\PLOSONE%20resubmission\8bb2132d-6a63-4231-a2d1-781145ab8097)

I don’t see why they can’t put it… you have the vegans, they’ve got the V, so perhaps if they had some sort of symbols or something next to it so you can see from there because at the bottom of their menu it says what it all stands for, whether it’s vegetarian or … I think it’s got peanut allergies on the one in (restaurant chain), and they’ve got all different symbols, and the symbols then are down the bottom. I think that’s fine

[<Internals\\P15 G2 S>](file:///F:\Food%20allergy%20&%20intolerance\Papers\Paper%201%20submission\PLOSone\PLOSONE%20resubmission\fae4c6b7-7de5-4193-9ed1-78119b642ed2)

We have a menu and then go up. I guess it would be the same, if you had a central list you could ask on the phone or when you go in. But the main menu that you get delivered has this sort of key, dairy free or gluten free, named ones….

[<Internals\\P3 G1 E>](file:///F:\Food%20allergy%20&%20intolerance\Papers\Paper%201%20submission\PLOSone\PLOSONE%20resubmission\95aa43c7-85a3-4449-81d1-780fbc892c66)

Just to have a little mark or little thing just to say oh yeah it does contain, you know.

[<Internals\\P9 G1 NI>](file:///F:\Food%20allergy%20&%20intolerance\Papers\Paper%201%20submission\PLOSone\PLOSONE%20resubmission\45f12a10-9e9e-4aba-82d1-7810e482f550)

You know how things say ‘contains nuts’, say there was a colour coded, and the red dot meant it contained wheat, one containing dairy. Those sorts of things, you’re not going to have to write out a list of ingredients because for some places that just would not be viable. You’d be reading 20 pages of a menu like a wine list. So some sort of colour code, like on toothpaste, like the square coding on the back of the tooth paste? That would be a system that would say ‘there’s dairy in that, stay away from that’. And if there’s just the lactose and no dairy in it or if there was just the dairy, those sort of things, a wee colour coded system.

**Separate menu / menu section**

[<Internals\\P15 G2 S>](file:///F:\Food%20allergy%20&%20intolerance\Papers\Paper%201%20submission\PLOSone\PLOSONE%20resubmission\fae4c6b7-7de5-4193-9ed1-78119b642ed2)

We have a menu and then go up. I guess it would be the same, if you had a central list you could ask on the phone or when you go in. But the main menu that you get delivered has this sort of key, dairy free or gluten free, named ones….

[<Internals\\A11 G1 S>](file:///F:\Food%20allergy%20&%20intolerance\Papers\Paper%201%20submission\PLOSone\PLOSONE%20resubmission\34e7524f-e359-45ca-99d1-780a919588c7)

I’m so used to looking on the back of products and being able to go, okay, well, that’s dairy, that’s…you know…and some places will even obviously have it in a separate section, you know, and that’s really handy. Restaurants are generally pretty bad.

[<Internals\\A31 G1 S>](file:///F:\Food%20allergy%20&%20intolerance\Papers\Paper%201%20submission\PLOSone\PLOSONE%20resubmission\78b2500b-e00b-46f7-bcd1-780c7b7a3a64)

I mean, it would be just much better if every ingredient was labelled there on the menu, or on a separate sheet to the menu and they can bring it to you and you can have a look for yourself. I’d rather that than bothering other people with it.

[<Internals\\A36 G2 W>](file:///F:\Food%20allergy%20&%20intolerance\Papers\Paper%201%20submission\PLOSone\PLOSONE%20resubmission\59593836-f838-45b1-a2d1-780cecdee7f1)

Maybe have a section where you can have something to eat without…telling you dairy-free, gluten-free, or whatever, yeah.

[<Internals\\A45 G1 W>](file:///F:\Food%20allergy%20&%20intolerance\Papers\Paper%201%20submission\PLOSone\PLOSONE%20resubmission\e2994b15-8cd8-45ee-9cd1-780d9d23ad3e)

But if they did, put underneath what the, you know, think of what people’s allergies could be and say that there is this, that or the other that is in there, you know what I mean, and people would know then to avoid it, you know.

[<Internals\\A46 G2 E>](file:///F:\Food%20allergy%20&%20intolerance\Papers\Paper%201%20submission\PLOSone\PLOSONE%20resubmission\c84dee5c-69a7-491e-a0d1-780db538d920)

Yeah, it was a book, em, of all their dishes, everything – like it would have, I don’t know, so-and-so, and then each component of the dish and whether it had any allergens or anything like that in it. So, I was able to go down and see, so I had a choice, which was lovely, of what I could have, and that was really helpful. I haven’t been back – obviously don’t live there.

[<Internals\\A56 G1 E>](file:///F:\Food%20allergy%20&%20intolerance\Papers\Paper%201%20submission\PLOSone\PLOSONE%20resubmission\25a9cd1a-0d74-4321-85d1-780e876b9cb9)

It would be amazing if people could have just an extra page on the menu that you could just go to because when you go into a restaurant, sometimes… I mean, I was hopeless before. I couldn’t, you know, make a decision before I knew there was anything wrong, when I could have everything, or I thought I could, but they’re so vast, so you’re faced with a vast amount of choice, which you think, you know, what do I want, ooh, I want that, I want that, I want that, and then you think can I have that, and then it goes. If there was just an extra page that just had, you know, like “Safe for weirdos” [laughing], a page at the back, or they had an allergy board….it doesn’t have to be all-singing, all-dancing. It’s just…just information, em, with like a little paperclip or something just on the top, just so that you, you know, you’re not taking the time to say it and say it and say it.

[<Internals\\A57 G2 E>](file:///F:\Food%20allergy%20&%20intolerance\Papers\Paper%201%20submission\PLOSone\PLOSONE%20resubmission\2cfe0d8b-6bd8-498d-88d1-780ea256cc44)

We were in (city), in a restaurant there, and they produced a gluten-free menu, which I thought, wow, yeah! That was the (restaurant name), in (city). There, they actually had their own personal gluten-free menu, which was wow!

[<Internals\\A8 G2 E>](file:///F:\Food%20allergy%20&%20intolerance\Papers\Paper%201%20submission\PLOSone\PLOSONE%20resubmission\c6261951-47fd-4b41-8ed1-780a57fd7dc5)

Because I know, em, actually, at (coffee shop chain), they started to introduce a menu that had the different things in and the intolerances, but I don’t know where that’s gone now, because it was, for a few months, they started to have it in – they had all the calories, and they had how much dairy in, whether it was vegetarian, whether it was whatever, and that was at (coffee shop chain). It was a foldout menu, but I haven’t seen it now for [a good year].

[<Internals\\P11 G1 W>](file:///F:\Food%20allergy%20&%20intolerance\Papers\Paper%201%20submission\PLOSone\PLOSONE%20resubmission\3b0e3dac-a9d4-4eae-a7d1-7811234b404e)

If it was plain and simple, if you could understand what they mean and things, I’m not saying it takes over the whole menu, but it is written in a small box or something at the bottom of the menu and explains whatever, you know I wouldn’t ask so many questions on the food, or so many questions in a restaurant, it’s bound to save some time and energy and whatever. But if it was more detailed, especially with the people who’ve got more severe allergies than (child’s name), especially the nuts and the strawberries and so on, especially for them…..I think it’d be brilliant. They’ve got little boxes here with the children’s choice and they should put allergens, a little box like that.

[<Internals\\P12 G1 W(51)>](file:///F:\Food%20allergy%20&%20intolerance\Papers\Paper%201%20submission\PLOSone\PLOSONE%20resubmission\8bb2132d-6a63-4231-a2d1-781145ab8097)

**….**we know the menus and it’s easier to find. Because we’re used to the menus, you know, you get used to just looking for the allergy section….It’s similar to the big one but it’s a lot smaller and it’s got the vegans and it’s just got the milk intolerance. It’s got everything on them lines, but it’s half the size of their normal menu, so the variety is not as good. It is good, but (child) orders the same thing every time.

[<Internals\\P3 G1 E>](file:///F:\Food%20allergy%20&%20intolerance\Papers\Paper%201%20submission\PLOSone\PLOSONE%20resubmission\95aa43c7-85a3-4449-81d1-780fbc892c66)

On the – yeah on the menu. If you order a take away either on line or on the menu if you have one obviously from the other take aways. I mean yeah I suppose on the menu, just underneath. You know whatever they’re sort of stating they could just have a little section. I think some of them do.

[<Internals\\P5 G2 E>](file:///F:\Food%20allergy%20&%20intolerance\Papers\Paper%201%20submission\PLOSone\PLOSONE%20resubmission\a051d7cb-be9c-4186-bcd1-781060f3071b) - § 3 references coded [1.05% Coverage]

So I asked them if they did anything and they actually came out, the waiters and waitresses have got a book a bit like what you’re holding there, everything listed and what’s in it. And they can actually say ‘yes that hasn’t got that in’.

[<Internals\\P6 G1 S>](file:///F:\Food%20allergy%20&%20intolerance\Papers\Paper%201%20submission\PLOSone\PLOSONE%20resubmission\c86011b3-5347-424b-84d1-781079ecb721)

With (restaurant chain) again they have a very good junior section, a very detailed menu, they are very family orientated, it can be a bit noisy, especially in (restaurant chain), sometimes it’s families galore!

**Positive interactions with staff**

[<Internals\\A12 G3 S>](file:///F:\Food%20allergy%20&%20intolerance\Papers\Paper%201%20submission\PLOSone\PLOSONE%20resubmission\b7ba2429-fb15-41e1-afd1-780aa00fc7ed)

I would ask the waiting staff, and if they don’t know, I would expect them to ask the chef. A lot of them do say “Oh, I’m not sure about that, I’ll ask the chef for you.” Others are quite, you know, they’re quite knowledgeable and they know what’s in it and they explain, especially in the likes of, say, (coffee shop chain) in (place name), the (restaurant). All their waiting staff all know what’s in what.

[<Internals\\A13 G2 W>](file:///F:\Food%20allergy%20&%20intolerance\Papers\Paper%201%20submission\PLOSone\PLOSONE%20resubmission\b02abc14-81eb-47b9-a7d1-780aaff3d798)

I think I feel most comfortable, definitely, when… Like I went to (pizza restaurant chain) this weekend and they give you a separate slate that’s different to everyone else’s pizzas and your own cutter, so then I know…I know that it’s fine because I’m doing it myself. And I just panic like…just the silliest things, like what if they give my gluten-free dish to someone else and give me theirs!

There was a bit of…she had a reaction because they’d given her something wrong. So, that as bad, but then she emailed and told the manager about it and they said, right, they apologised profusely, and were like, when you come back, tell us you’re coming back and we’ll make sure your experience is much better. We actually went back about two weeks ago, and she explained this is me from before, bad experience, and you told me to let you know if I was coming back – I’m back. And it was like, right, and there was a huge wait for tables, but they gave us a table straightaway. We got to speak to one of the higher staff, like with the manager, who knew more about it. She took our order and she was like, right, we’ll make sure there is no cross-contamination. She mentioned that, which was a big…you know, they sent over a waitress who had an experience herself and knew a family member with it, so then obviously we felt more comfortable because we were speaking to someone who’d experienced it themselves. So, that was good.

[<Internals\\A14 G2 S>](file:///F:\Food%20allergy%20&%20intolerance\Papers\Paper%201%20submission\PLOSone\PLOSONE%20resubmission\d0ec6497-eed6-4f58-a3d1-780abfa84ba4)

(Supermarket deli). The food, I know, is made fresh, and they ask me – this was the part, they ask “Do you want cheese on that?” No. They ask you. They don’t ask you out of kindness, they ask you because they charge you more for it, but it’s the fact they ask me, and they tell me if…not because [?], but if, for example, they’ve run out of butter or margarine, they’ll say “Oh, we’re using margarine instead of butter,” or… They’re quite specific at this one.

[<Internals\\A19 G2 NI>](file:///F:\Food%20allergy%20&%20intolerance\Papers\Paper%201%20submission\PLOSone\PLOSONE%20resubmission\e9119547-10cd-43a4-81d1-780b6d6a5dab)

And then the (name) Hotel in (place name), I was at a wedding there and told them I was gluten-free, and then, when I arrived, the man came over and asked me if I was the gluten-free, because the bride had pointed me out, and then he got me a nice starter and things, and the dinner and offered me pavlova for dessert and things, apart from fruit, because I said I couldn’t have fruit, so they dealt with that quite well.

[<Internals\\A2 G1 E>](file:///F:\Food%20allergy%20&%20intolerance\Papers\Paper%201%20submission\PLOSone\PLOSONE%20resubmission\f3242133-96f9-427d-81d1-7809db751f8f)

So I did go and – when he came over, “Do you have that?” and he was like “Oh, I’m not sure – to be honest with you, sometimes it seems we do and sometimes we don’t,” and I imagine it happens like that in a kitchen. But he went and spoke to the chef and just made perfectly sure or said “Would it be okay not to do it?” and he even came back with more information than needed. He went, “Oh, she can’t guarantee it hasn’t touched…been in a nut environment, and she says, you know…” blah-blah-blah, which is fine because he could have just went, “No, it’s not coming with nuts.” He went the extra mile and he was actually clear and I was, oh, that’s fine, that’s fine, and I got my cake! So, yeah, that was very good.

[<Internals\\A21 G3 NI>](file:///F:\Food%20allergy%20&%20intolerance\Papers\Paper%201%20submission\PLOSone\PLOSONE%20resubmission\99c18223-cce9-4231-aad1-780b970daf7e)

Yeah, because there’s plenty of times we’ve said that and they’ve come over and said, right, such-and-such, you know, it’s been done that way – is that okay for you?

[<Internals\\A28 G1 W>](file:///F:\Food%20allergy%20&%20intolerance\Papers\Paper%201%20submission\PLOSone\PLOSONE%20resubmission\df9f8d1a-9ed7-4cf7-96d1-780c3af72ff1)

They’ll tell you what to avoid, or they’ll cook it from scratch for you, which I’ve known to…it has been done….Yes. You know, they don’t take offence when you ask! Most of them are quite nice, but they know.

[<Internals\\A3 G3 E>](file:///F:\Food%20allergy%20&%20intolerance\Papers\Paper%201%20submission\PLOSone\PLOSONE%20resubmission\284cfbd0-b520-4716-80d1-7809ec89df6c)

Em, well, if you ask them and they…they’re pretty helpful, not…not over-the-top helpful, but if they don’t know, they’ll say, “Oh, I’ll just check.” You know, like with the (Asian restaurant), you know, “It’s not done with yoghurt, is it, and it’s not done with cream?” and they say, you know, no, and, “Oh, hang on a minute…” and what makes – I do like it if they say, “Oh, hang on, I’ll just check.” Because they could say, “No, it’s not,” you know, and I think, well, if they’re just checking, they are taking an interest…

[<Internals\\A31 G1 S>](file:///F:\Food%20allergy%20&%20intolerance\Papers\Paper%201%20submission\PLOSone\PLOSONE%20resubmission\78b2500b-e00b-46f7-bcd1-780c7b7a3a64)

These sort of places, I would…I would tell them at the beginning because I know that the service is so good and they’re so sort of knowledgeable about their food that it wouldn’t be an issue at all, and they’re really just happy to…to help.

[<Internals\\A38 G3 W>](file:///F:\Food%20allergy%20&%20intolerance\Papers\Paper%201%20submission\PLOSone\PLOSONE%20resubmission\909439ae-b553-41c3-9fd1-780ef23fbe1f)

I think, because I’ve been going, you know, I’ve been going to, you know, like the [venue name], I’d been going there because I became intolerant, so I know the staff and things, so I know that they will cater… If I say, “Oh, please,” you know, they’ll be like “Of course!” So, I don’t feel that I’m sort of putting them out or anything because I sort of…I know them on a personal level.

[<Internals\\A39 G1 W>](file:///F:\Food%20allergy%20&%20intolerance\Papers\Paper%201%20submission\PLOSone\PLOSONE%20resubmission\7f01cf85-a7f7-47a8-9bd1-780d2352613c)

(Sandwich chain) are usually quite good because I’ve been…went to one years a couple of years ago now, and I said, “Oh can I have that, but I’m allergic to …. so you’re going to have to completely…” you know, and she said, “Well, that’s cut in the same machine, so you can’t have that.” So, they kind of know what’s…what’s cut what and what’s doing what. So, Subway are quite good for knowing what’s in the products and stuff.

[<Internals\\A4 G2 E>](file:///F:\Food%20allergy%20&%20intolerance\Papers\Paper%201%20submission\PLOSone\PLOSONE%20resubmission\91904adb-2d54-4a23-b0d1-780a005a7498)

But if someone was proactive in sort of saying, you know, here’s the menu today, we’ve got this, we’ve got that, and it’s got a gluten-free section, it may make those people with gluten-free…oh, fair enough, that’s quite good, I’ll look at that. Em…so I suppose the confidence of knowing what’s in the food and how it’s prepared and all that sort of stuff, I suppose…

[<Internals\\A40 G1 W>](file:///F:\Food%20allergy%20&%20intolerance\Papers\Paper%201%20submission\PLOSone\PLOSONE%20resubmission\922ffa83-21ec-43ad-bbd1-780d35f24675)

I’ve found that they’ve been really helpful. If they aren’t sure, they usually do go and ask. They usually ask the cook.

[<Internals\\A41 G1 W>](file:///F:\Food%20allergy%20&%20intolerance\Papers\Paper%201%20submission\PLOSone\PLOSONE%20resubmission\e6fb7a1f-62ea-4c0a-86d1-780d5002770a)

If they looked like they were enjoying their jobs and they looked like… If was someone was just like saying to you like, “Oh yeah, what do you want to order?” or do they look like they actually wanted to serve you and wanted you to ask questions really…

[<Internals\\A43 G1 S>](file:///F:\Food%20allergy%20&%20intolerance\Papers\Paper%201%20submission\PLOSone\PLOSONE%20resubmission\b78f1290-ec1b-4e9b-90d1-780d7006196d)

As I said, because I find, if you just ask, they seem to change things for you, most places.

[<Internals\\A45 G1 W>](file:///F:\Food%20allergy%20&%20intolerance\Papers\Paper%201%20submission\PLOSone\PLOSONE%20resubmission\e2994b15-8cd8-45ee-9cd1-780d9d23ad3e)

I might say “Is there cream with that? Is there custard with it?” is there this, is there that, you know, what… I’ve even had them come up, bringing a piece out to me to show me

[<Internals\\A49 G1 E>](file:///F:\Food%20allergy%20&%20intolerance\Papers\Paper%201%20submission\PLOSone\PLOSONE%20resubmission\045461f6-fb51-4d35-bbd1-780dec8c15be)

“Oh, but can I have this, but not with this?” or, you know, something, that I have these kind of special requests, that it’s just like “Em, okay then…” You know, I like to be like “Of course, absolutely!” If they are like very open to that, then I think that’s somewhere, you know, I enjoy – I have an enjoyable experience being at.

[<Internals\\A5 G2 E>](file:///F:\Food%20allergy%20&%20intolerance\Papers\Paper%201%20submission\PLOSone\PLOSONE%20resubmission\f46b0bee-aff2-4a4b-9fd1-780a1c7fd9ad)

Yeah, I would ask them, but I…depending on how they react, their answers, is how much I believe them, because, you know, you can say does that have something in it and they go “Oh no!” or “Oh yes!” and they know, but if they say, “Oh, I don’t know, I’ll have to check with the chef,” and all that, you think, well, you should know the answer to that really, if you work here, you know. So, em, yeah, I’d always ask them and then see what their answer is, if I trust them or not

[<Internals\\A50 G2 E>](file:///F:\Food%20allergy%20&%20intolerance\Papers\Paper%201%20submission\PLOSone\PLOSONE%20resubmission\d16fd7e9-55c8-4f01-bed1-780e03a28ebb)

I’m probably more prepared to take the word of the food provider. I mean, an example of that was, on Saturday, we went up to the contemporary craft festival, and there was a vegan/vegetarian stall there, and she said “Everything here is gluten-free, except for the cakes in this…some of the cakes here.” And I thought, oh, that’s great…So kind of…yes, knowledge that’s demonstrated by their people in the café or restaurant or whatever it might be.

I think the provenance of food, the integrity and the authenticity of food is…it’s mattering to people I think a lot more. So I think that’s something that also informs my sense of whether I trust somebody or not. So, if I can go in and…like…a little bit like that place at the craft festival on Saturday, you know, I think I bought into her, my sense of her integrity about what she was doing, that she seemed knowledgeable, she seemed, you know…

[<Internals\\A52 G1 S>](file:///F:\Food%20allergy%20&%20intolerance\Papers\Paper%201%20submission\PLOSone\PLOSONE%20resubmission\67ce6f99-70f0-48eb-96d1-780e28ca8861)

… you know, the kind of place where you know exactly that the chef is going to be able to do what it is that you want or help you avoid something, and where the waiter is going to know what – you know, if you say “Is there something in this?” he’s going to know and not have to sort of shuffle off and ask, you know, sort of supervisors and things.

I was looking for something really un-spicy, you know, was staying away from wheat and things, and they said, you know, “Don’t worry, you know, we’ll make it as spicy as you want, and if you say you just want mild, mild, mild, that’s what we do,” kind of thing, so, “And there’s no wheat in any of our sauces” and things, so, em, yeah, that would be probably the type of place I would go.

[<Internals\\A56 G1 E>](file:///F:\Food%20allergy%20&%20intolerance\Papers\Paper%201%20submission\PLOSone\PLOSONE%20resubmission\25a9cd1a-0d74-4321-85d1-780e876b9cb9)

I was out with my mum, and she had said, you know, we’ll go for a scone, and I was like, oh, I can’t have a scone, and I just said, “Oh, it doesn’t matter, I’ll have whatever,” a biscuit or whatever, and then she said, “You know, these don’t look like they’re glazed mind, because they’re just really pale,” and she said, “Oh well, I’ll ask,” and I was like, “Oh, you know, don’t bother,” but she did, and they said, “Oh, no, no, we don’t glaze them. We do with the cheese ones, but not the fruit ones – there’s no egg at all in them or on them,” so total result!

Yeah, they’re really good because they just…there’s a little note under everything on their shelf, or you can see through it so you can see if there’s eggs on the top or… But they’re great like that, and normally, as well, their staff don’t have to go and look – they know because they make them out the back, so they know straightaway.

…you can see the chefs, and they’re totally accessible to just nip up and say “Excuse me, do you bind the burgers with egg or not?” you know, and they’d say “No, we don’t, but the breadcrumbs on the chicken are, but we can do them without if you want.” And it’s just like that quick – you don’t have to ask a waitress to go in the back to ask two or three chefs to come out, so that makes a difference.

[<Internals\\A59 G1 S>](file:///F:\Food%20allergy%20&%20intolerance\Papers\Paper%201%20submission\PLOSone\PLOSONE%20resubmission\d8a0face-1668-42ec-8ed1-780ec9326302)

I’ll go, and I just said “I have an allergy, will it be okay?” and they were like “Yeah, and we’ll keep it in a separate – there’s a separate [pot/part], there’s a separate area, and it will be prepared on a separate board. It will be fine.” And, yeah, so it was, and any of the dishes that actually have nuts in them that I wanted, they said that they could prepare separately so I could eat them, so they were really, really good there.

Yeah. You see, that was very good. That was a rarity. I wouldn’t…I didn’t expect it to be that good when I went. Everywhere has kind of just got this…”Oh well, we can’t guarantee it,” because they don’t want to be in trouble if I do have a reaction. So, everywhere is kind of the same really.

[<Internals\\A6 G1 E>](file:///F:\Food%20allergy%20&%20intolerance\Papers\Paper%201%20submission\PLOSone\PLOSONE%20resubmission\37f504f9-f51f-4f42-93d1-780a2e934c5a)

I suppose just the thing that you’d got to know them and stuff. Like, going in somewhere new, asking a waitress “Is there nuts in this?” and getting a “No” because they don’t think there is, but they haven’t asked the chef, whereas someone where I’ve been in, and every time I go in, I might ask, and they’re like, “No (name of interviewee), there’s no reason there should be nuts in it today!” you know, sort of like just used, yeah, just used to it. They do do food with nuts in, but I’ve spoke to them in the (Asian) shop and they’re not prepared in the same places, so they’re fine, like as far as they’re concerned, and obviously I’ve taken her word on that occasion and tried it, and never had a problem. It’s really hard because I love that food, but I do have to be careful.

[<Internals\\A60 G1 E>](file:///F:\Food%20allergy%20&%20intolerance\Papers\Paper%201%20submission\PLOSone\PLOSONE%20resubmission\0512df0d-473a-4251-b5d1-780edadf7bd5)

The ones we tend to frequent [in] bits and pieces, em, I’ve actually gone in – they’ve taken me into the kitchen and shown me that they do keep separate containers and bits and pieces, and they don’t have nuts just sat around.

… if you get brushed off really: “Oh yeah, yeah, we can deal with that, that’s absolutely fine – oh yes, of course it’s nut-free, “ or “Of course it’s wheat-free.” There’s no substance to that whatsoever. Whereas, if somebody actually takes the time to come out and say, you know, yes, this is…or “We can guarantee that this is…” you know, “We keep everything in separate containers and [there’s no] cross-contamination…”

[<Internals\\A8 G2 E>](file:///F:\Food%20allergy%20&%20intolerance\Papers\Paper%201%20submission\PLOSone\PLOSONE%20resubmission\c6261951-47fd-4b41-8ed1-780a57fd7dc5)

If I’ve asked, em, for them to go and say, “Oh, I’ll go and ask,” rather than me having to say “Can you go and ask for me?” or them to just know or to have…

[<Internals\\P1 G1 E>](file:///F:\Food%20allergy%20&%20intolerance\Papers\Paper%201%20submission\PLOSone\PLOSONE%20resubmission\a7d7d4cc-b730-4b1a-a0d1-780f8eb1ec4b) -

Looking at the menu but because it’s quite an expensive restaurant the people who serve you are very knowledgeable, they would do anything to sort of help you so I find that the more you pay, the better the service. So then you can find out much more information they can tell you exactly how things are cooked, exactly. So it’s safer from that option because you know what is a definite no, no.

To me some people I think seem to be more sympathetic than others and then you don’t want to go back to that place. But that is how I’ve found it.

[<Internals\\P2 G1 E>](file:///F:\Food%20allergy%20&%20intolerance\Papers\Paper%201%20submission\PLOSone\PLOSONE%20resubmission\9fde6335-bd8c-4ecb-a0d1-780fa28280aa)

When I talked to the frontline staff, some were a little unsure, some would say let me get the manager for you, or some would wait for me to say, “Can I talk to the manager?” None were unpleasant; they want you to come back, so there has to be some kind of mediation. Most of the managers seemed aware of what products they were using, some could say pretty much off the top of their heads what kind of bases they were using. Some who were very good said let me double check. To make sure, some would say no it’s not in it.

… sometimes you get a lot of young counter staff, no disrespect to them, but they are just sales staff. Then you get older staff, who know a little bit more, or who’ve been there longer, who know a bit more about the ingredients within the products. It seems to be an age thing, the older the member of staff, the more kind of proactive….

[<Internals\\P5 G2 E>](file:///F:\Food%20allergy%20&%20intolerance\Papers\Paper%201%20submission\PLOSone\PLOSONE%20resubmission\a051d7cb-be9c-4186-bcd1-781060f3071b) - § 3 references coded [1.05% Coverage]

They can fob you off. But sometimes waitresses will go in and come back out and say ‘yep, the chef says this and this’ and it sounds quite positive. You sort of get more confident from other people’s confidence.

[<Internals\\P9 G1 NI>](file:///F:\Food%20allergy%20&%20intolerance\Papers\Paper%201%20submission\PLOSone\PLOSONE%20resubmission\45f12a10-9e9e-4aba-82d1-7810e482f550)

A good restaurant will also tell their, chef will also tell their servers, if people are allergic to nuts, fish, anything like that, this dish has it, this dish has it and compile a sort of list. If they don’t know these things then they should be able to go and ask the chef.

**Negative interactions with staff**

[<Internals\\A11 G1 S>](file:///F:\Food%20allergy%20&%20intolerance\Papers\Paper%201%20submission\PLOSone\PLOSONE%20resubmission\34e7524f-e359-45ca-99d1-780a919588c7)

So, I started eating this meal, and all of a sudden, it was like, um, I’m not sure about this…this isn’t a flavour or a texture or a shape that I’m very comfortable with here… The manager asked him and he was like, “No, no, it’s fine, it’s fine.” And I actually, I was so unsure of myself that I packed a couple of prawns into a napkin and went to another restaurant, like just up the road, and went “Are these prawns?” and they’re like, “Yeah!”, and I was pretty furious.

[<Internals\\A13 G2 W>](file:///F:\Food%20allergy%20&%20intolerance\Papers\Paper%201%20submission\PLOSone\PLOSONE%20resubmission\b02abc14-81eb-47b9-a7d1-780aaff3d798)

I suppose just their attitude towards it I think. Like both me and my sister have been made to feel really uncomfortable in a place before, and that’s been at the cost of us going in there again.

Just that…once, a waitress came over…we’d asked for… We asked for the gluten-free menu, and she was like, “Yeah, no problem,” brought them over to us and was like, “Okay, so which one of you are the awkward ones then?” and we were like, actually, we’re not being awkward, it’s a condition! We thought that was quite rude, and em, yeah, just being spoken to like that

[<Internals\\A19 G2 NI>](file:///F:\Food%20allergy%20&%20intolerance\Papers\Paper%201%20submission\PLOSone\PLOSONE%20resubmission\e9119547-10cd-43a4-81d1-780b6d6a5dab)

So, I just think that they don’t really…they don’t really know about it, and they can’t really… They ask me what I would like instead of telling me options that I could have that are gluten-free, so they don’t know either.

[<Internals\\A23 G2 NI>](file:///F:\Food%20allergy%20&%20intolerance\Papers\Paper%201%20submission\PLOSone\PLOSONE%20resubmission\d68a40bb-95a9-409b-96d1-780bbb80c1eb)

And then I also do sometimes believe that, from my own experience of working in retail, a lot of the time, I think if you ask somebody, you know, in a restaurant, a waiter, “Is there corn flour in that?” they’ll probably just say no or yes on the spur of the moment, without even knowing, just to tell you something

[<Internals\\A24 G1 NI>](file:///F:\Food%20allergy%20&%20intolerance\Papers\Paper%201%20submission\PLOSone\PLOSONE%20resubmission\ac115d93-00c6-47cc-b8d1-780beaf8d184)

Well, I was in, em, I was in (burger chain) once and I asked for a burger. I asked for a plain one and it came with salad on it. Whenever I took it back, it was just scraped off, you know, so I could still see that, you know, there was still some of it on.

[<Internals\\A25 G1 E>](file:///F:\Food%20allergy%20&%20intolerance\Papers\Paper%201%20submission\PLOSone\PLOSONE%20resubmission\36cc6cd9-6f03-4df2-94d1-780bfedc702d)

They’ll say things like “Mm, we can’t be sure,” and things like that, and it could be in contact with nuts.

Em…maybe if there’s more young staff, they’re probably new and they don’t know much about the kitchens, yeah, that would put me off a little bit.

[<Internals\\A27 G1 W>](file:///F:\Food%20allergy%20&%20intolerance\Papers\Paper%201%20submission\PLOSone\PLOSONE%20resubmission\0264b4c7-aa60-43a7-aed1-780c25a4fc11)

Yes, and you can ask them. Well, in (Asian restaurant) and these restaurants, you can ask them and they will say “Oh no, there’s no peanuts in it,” and you see them and you know they’re…They don’t tell you the truth, do they? No. Because, after all, they’re in the game to make money, they want to sell their food, doesn’t matter about you…..Yes. You can’t blame the waitresses, in a way, because they don’t know what’s in the things, do they?

[<Internals\\A31 G1 S>](file:///F:\Food%20allergy%20&%20intolerance\Papers\Paper%201%20submission\PLOSone\PLOSONE%20resubmission\78b2500b-e00b-46f7-bcd1-780c7b7a3a64)

In (place name) – it was horrible! Me and my sister were camping, and there was a little café and this horrible woman… I went in and I could see there were nuts in lots of stuff, and I wanted a cake and I said “Has that one got nuts?” and I said okay, because it looked nutty to me, looked a bit nutty, and then… So she said, “No, no, it’s not, no!” and she was quite rude, and so I said, “Oh right, okay,” I said, “because I have got a serious allergy.” And this is really unusual for me to say that much, but she was not nice. So, she said, “Oh no, it’s definitely not – you know, I’ve told you it hasn’t.” So, I took it, sat outside on the bench, and one sort of taste or bite sort of said that, yeah, this has got almonds in it, and my lips went up. With the nut allergy - I didn’t develop anaphylaxis that time, it was just the kind of beginnings - you get like this sort of sense of doom, and that’s like one of the, em, sort of symptoms of anaphylaxis coming, and it’s horrible because you’re just like crying and you’re like I’m going to die, I’m going to die, and that’s the feeling – it’s horrible! So my poor sister was there and she was like, “Oh no!” you know, “What’s happening?!” and she sort of ran back in and she said, “That has got nuts in it!” and she checked then and said, “Oh right, yeah, okay…” but obviously when I was like… I was ill, but thankfully not to the extent I was last time.

[<Internals\\A32 G2 S>](file:///F:\Food%20allergy%20&%20intolerance\Papers\Paper%201%20submission\PLOSone\PLOSONE%20resubmission\1310ff29-9ef0-44b1-a1d1-780c8f995c68)

I’ve actually got a friend who, when I was telling them about the diagnosis, years and years ago, he used to work in a kitchen, and he had said a lot of people would, you know, the waiting staff would come through and say, oh, the person was asking is it gluten-free, and they used to just kind of laugh and say, “Oh, just tell them it’s not.” You know, I think a lot of people didn’t know that it is a serious thing, and that’s…that’s just, you know, a misconception, that people are thinking that, oh, they’re just trying to, you know, create a fuss or, you know, create a big problem.

[<Internals\\A33 G1 S>](file:///F:\Food%20allergy%20&%20intolerance\Papers\Paper%201%20submission\PLOSone\PLOSONE%20resubmission\b10d29da-9330-488b-83d1-780cb2c3f056)

I get it quite a lot on aeroplanes because they’re so busy trying to get people in the door and then taking off, and then, once you’re up in the air, that’s when they tell you. Often, that’s quite a hassle because what if somebody in the aeroplane is eating peanuts while taking off, and they never seem to remember, or they never seem to be that bothered about it. They tell you once [everybody is in] the aircraft, so that’s when I get quite riled, quite annoyed about the fact they’ve not told anybody yet, because it’s such an enclosed area, and you’re sharing the same air as everybody, another 500 passengers, so if somebody’s eating peanuts in the back of the plane, that air is all through, circulating where you’re sitting. [?].

[<Internals\\A34 G2 W>](file:///F:\Food%20allergy%20&%20intolerance\Papers\Paper%201%20submission\PLOSone\PLOSONE%20resubmission\e6390bbf-351c-4055-a2d1-780cc4ef315d)

I’ll send it back. If it comes and it’s not…I have done that, and I’ll just say that…just take it back. You know, like I’ve had salads and they’ve got dressings on, and most of them have got sesame in. You know, you’ll always get a sesame seed dressing, and if they’ve drizzled like that, I’ll say I can’t eat it. And you’re looking at £8 or £9…for me to push to the side….

[<Internals\\A36 G2 W>](file:///F:\Food%20allergy%20&%20intolerance\Papers\Paper%201%20submission\PLOSone\PLOSONE%20resubmission\59593836-f838-45b1-a2d1-780cecdee7f1)

Because I don’t think they’d have a clue. I couldn’t trust them. I think…they might have gone back to the kitchen and think, “Oh, I can’t be arsed,” and then come back and say, “Oh no, it’s fine.”

[<Internals\\A38 G3 W>](file:///F:\Food%20allergy%20&%20intolerance\Papers\Paper%201%20submission\PLOSone\PLOSONE%20resubmission\909439ae-b553-41c3-9fd1-780ef23fbe1f)

And it’s sort of like, well, you know, this is…has been asked… So, I do think that some places… But I think the majority of places now seem to be better, but I don’t know whether it’s the waiting staff that aren’t relaying the messages to the kitchen, I don’t know.

[<Internals\\A39 G1 W>](file:///F:\Food%20allergy%20&%20intolerance\Papers\Paper%201%20submission\PLOSone\PLOSONE%20resubmission\7f01cf85-a7f7-47a8-9bd1-780d2352613c)

Some places, (pub chain), you know, you go there for a quick bite and stuff – they’re rubbish. They write it all on the computer and it comes out on a plate, celery or something or cucumber. So, I think you learn to know where you can…..It’s the uncertainty of not knowing that people know what they’re doing. I know it sounds stupid…

I was eating it and I said – and we’d checked before, there’s no…? “No, it’s fine.” I took a mouthful and I was like “There’s something not right in there.” So, everyone else had a taste and it was like, “Tastes like there’s almonds in it…” I was like, oh [sighing], and of course it started coming up again and… So, they came out and were like, “Oh, so sorry, so sorry!” but it was all planned in advance so it should have been… It was a mistake on the waiter’s part that…

[<Internals\\A44 G1 E>](file:///F:\Food%20allergy%20&%20intolerance\Papers\Paper%201%20submission\PLOSone\PLOSONE%20resubmission\f74714b6-db56-491b-bed1-780d8cb32ccf)

If they show me or they actually physically get the item and bring out the packaging, whatever, which I’ve had, you know, then it’s…then it gives you that level of confidence. But if they go “Oh, I’m not sure what that means,” or “Oh, I don’t know,” you know, you can just tell by that person [laughing], and that’s when I’m like, no, don’t worry, I won’t eat it [laughing]!

And that’s what he kept coming out and saying to me, “Oh, I don’t know – we haven’t got that, we haven’t got that, you know, we don’t know the answers,” to some of them. But because he said it differently, it made me feel a little bit less angry, but because the girl behind the counter was going, “Oh, no, this is only how you can have it, no, this is only…”

[<Internals\\A46 G2 E>](file:///F:\Food%20allergy%20&%20intolerance\Papers\Paper%201%20submission\PLOSone\PLOSONE%20resubmission\c84dee5c-69a7-491e-a0d1-780db538d920)

You know, you just feel a bit, em, I don’t know, like you’re being a bit demanding or something when, you know, if those people could come and see the reaction that it’s going to have, then they probably wouldn’t be, you know, quite so judgemental.

[<Internals\\A48 G2 S>](file:///F:\Food%20allergy%20&%20intolerance\Papers\Paper%201%20submission\PLOSone\PLOSONE%20resubmission\92df34fb-0285-4959-83d1-780ddaefa70d)

It depends. If it’s an older person serving you, then yes, but if it’s a younger person, they go, “Oh, what’s coeliac…?!”

[<Internals\\A49 G1 E>](file:///F:\Food%20allergy%20&%20intolerance\Papers\Paper%201%20submission\PLOSone\PLOSONE%20resubmission\045461f6-fb51-4d35-bbd1-780dec8c15be)

Sometimes, yeah. Like, sometimes, em, they say, ”Oh, I’ll go to the kitchen,” and then come back and then they can’t really remember or something, and then they’ve said “Oh, I’ll just get the chef out to talk to you about…

Yeah, I think it is a thing that sometimes you will say like “Oh, but I’ll have to have it without the dressing,” and they bring it out and they’ve completely forgotten that you asked for that, and it’s like drenched in it, and you have to, you know, again, say, “Well, actually, I did say…can you make a new one?” which is a bit of a pain.

Whereas, some places, you know, it might be like the sauces, that I like the sound of the sea-bass and it’s coming with like a crayfish infused sauce or something, so obviously I can’t have that, but I want to have the sea-bass, like I find these places will whip you up some other kind of sauce that will be complementary, which is really good, but I find some places will just, “Okay, but you just won’t have any sauce.” You’re just like eating something bland and I find that like not as…

[<Internals\\A5 G2 E>](file:///F:\Food%20allergy%20&%20intolerance\Papers\Paper%201%20submission\PLOSone\PLOSONE%20resubmission\f46b0bee-aff2-4a4b-9fd1-780a1c7fd9ad)

Yeah, I would ask them, but I…depending on how they react, their answers, is how much I believe them, because, you know, you can say does that have something in it and they go “Oh no!” or “Oh yes!” and they know, but if they say, “Oh, I don’t know, I’ll have to check with the chef,” and all that, you think, well, you should know the answer to that really, if you work here, you know. So, em, yeah, I’d always ask them and then see what their answer is, if I trust them or not

[<Internals\\A50 G2 E>](file:///F:\Food%20allergy%20&%20intolerance\Papers\Paper%201%20submission\PLOSone\PLOSONE%20resubmission\d16fd7e9-55c8-4f01-bed1-780e03a28ebb)

A couple of times, em, I think there was one time I ended up with a plate of what was definitely pearl barley, and I just said “I’m sorry, I can’t eat that.” So, you know, the chef came out and it was like “But it’s barley, it’s not wheat!” Yeah! So, sometimes, there’s not an understanding about…you know, it’s not just about wheat, it’s other grains as well. So, I feel that, you know, it is my responsibility to be clear about what I’m eating, but also to, you know, be prepared to say “I can’t eat that” or educate.

I nearly got beaten up by a chef. It’s a long time ago. I must have been in my early-twenties and it was a fish meal – that’s all I can remember – and the query went back, you know, “Is this gluten-free?” and he came out and he was absolutely ranging, “Who’s asking about…?!” you know, di-di-di. And it was kind of…it was such an aggressive response to that request, em, I was quite shocked by it.

[<Internals\\A52 G1 S>](file:///F:\Food%20allergy%20&%20intolerance\Papers\Paper%201%20submission\PLOSone\PLOSONE%20resubmission\67ce6f99-70f0-48eb-96d1-780e28ca8861)

I tried to ask if there was any nuts in anything, and I got the blank look, you know, from the little girl who was serving us. I mean, you know, these…you know, they’re kids who are part-time. That’s, you know, they aren’t been paid enough to know. You know, they don’t sort of…they’re not close enough to the food to know. But, you know, then we got the supervisor, who then checked with the manager – you know, it was just…what a nonsense. And, yeah, I mean it was…especially when you’re with kids who are starving, you know, and want to get ordering and things, and it was busy anyway, and yeah, it was just…it was not the best experience.

[<Internals\\A59 G1 S>](file:///F:\Food%20allergy%20&%20intolerance\Papers\Paper%201%20submission\PLOSone\PLOSONE%20resubmission\d8a0face-1668-42ec-8ed1-780ec9326302)

Yes, sometimes they won’t… I like it when they go to the kitchen and they come back to the table and tell you that it’s fine, but a couple of the places I’ve been, they’ve not come back to the table, and then they’ve just brought the food over, and I’ve been like, “Oh, this is definitely okay?” and they’re like, “Eh, yeah…” So, I don’t know whether it’s just like the waitress or waiter is forgetting to go and ask the kitchen, but then I’m always a bit like ooh …I ask them to re-check in the kitchen and they come back and then I eat it. I wouldn’t eat it until they’ve checked

[<Internals\\A60 G1 E>](file:///F:\Food%20allergy%20&%20intolerance\Papers\Paper%201%20submission\PLOSone\PLOSONE%20resubmission\0512df0d-473a-4251-b5d1-780edadf7bd5)

I’ve been to restaurants before where you tell them you have an allergy and they still serve you up, you know… And you ask for, you know, “What’s in the sauce – are there any nuts?” Is there this, that or the other…. “Oh, no, no, no, you’re absolutely fine!” and, sure enough, there are….Because there’s other places, you call and they “Oh, yes, yes, we can cater for that!” and then there’s no substance to it whatsoever. I think, with people with severe allergies, you take a bit of a punt any time you go out, for any meal really. It’s as much about the interaction with the staff.

[<Internals\\A9 G1 E>](file:///F:\Food%20allergy%20&%20intolerance\Papers\Paper%201%20submission\PLOSone\PLOSONE%20resubmission\e36a7a38-30ff-44ec-b9d1-780a69f6c1df)

Actually, yeah, there was once, when I had a pasta with pesto, and pesto includes pistachios so it’s kind of nuts, yeah, so… [What I had] was…because it was…because we used to serve this meal like with little bowl of pesto, and she thought she can just take away that bowl, but it was a little bit, it dropped, so it was already inside the pasta. Yeah, so it was…that caused a little bit, mm…

[<Internals\\P1 G1 E>](file:///F:\Food%20allergy%20&%20intolerance\Papers\Paper%201%20submission\PLOSone\PLOSONE%20resubmission\a7d7d4cc-b730-4b1a-a0d1-780f8eb1ec4b)

I mean you try to ask, you try to look and see what is on the menu, you try to ask but you know it doesn’t always work perfectly. So sometimes the waiters and waitresses don’t know, sometimes I think they don’t even really understand what I’m trying to explain to them about lactose and what it is. So I don’t expect them to get it….

It’s amazing that some people who are waiting on tables don’t even know what lactose is or don’t really understand what it is in. So I think in terms of training in this day and age, if someone has got a gluten allergy or whatever they should know, the people serving the food should at least have some clue, so they can give some advice.

[<Internals\\P11 G1 W>](file:///F:\Food%20allergy%20&%20intolerance\Papers\Paper%201%20submission\PLOSone\PLOSONE%20resubmission\3b0e3dac-a9d4-4eae-a7d1-7811234b404e)

Yeah I’ve had a few places that they just think I’m a bit of a fussy mother probably. I can’t think of the names of them now. I know once at a carvery they did, a bit like cauliflower cheese, but it was broccoli, cauliflower and melted cheese, and I did say to the chef one day, the boy on the counter, because he was just throwing some roast potatoes in, and I said ‘Does that contain cheese?’, ‘Well what do you think?’ he went and he walked off. Well there is no menu to say there is broccoli and cauliflower with melted cheese on top, it just looked like roast potato on top of cauliflower cheese and it was just grilled. If I hadn’t have asked I wouldn’t have thought that was cheese on the top. I just thought it was crunchy potato put on the plate.

[<Internals\\P14 G3 E>](file:///F:\Food%20allergy%20&%20intolerance\Papers\Paper%201%20submission\PLOSone\PLOSONE%20resubmission\f7ffecf1-e304-4bd6-9ad1-78117b75f8a3)

Yes, but I haven’t done anything about it at the time, like this ice cream place where it wasn’t diary free and there was something else recently I said to (name) and I said this has got cream in it so you can finish it off

[<Internals\\P15 G2 S>](file:///F:\Food%20allergy%20&%20intolerance\Papers\Paper%201%20submission\PLOSone\PLOSONE%20resubmission\fae4c6b7-7de5-4193-9ed1-78119b642ed2)

And the only café we go to is in a family centre and they sell healthy food but the chef, I don’t know what he was thinking. I took my own soup in for lunch because it’s not always dairy free. And the guy was fine with us heating up our soup but then eh sort of said ‘our soup’s always gluten free and its always dairy free’. The assistant was behind and said ‘yeah but there’s parmesan in it today!’ Yes, that’s why I’ve got my own soup.

[<Internals\\P2 G1 E>](file:///F:\Food%20allergy%20&%20intolerance\Papers\Paper%201%20submission\PLOSone\PLOSONE%20resubmission\9fde6335-bd8c-4ecb-a0d1-780fa28280aa)

sometimes you get a lot of young counter staff, no disrespect to them, but they are just sales staff. Then you get older staff, who know a little bit more, or who’ve been there longer, who know a bit more about the ingredients within the products. It seems to be an age thing, the older the member of staff, the more kind of proactive….

[<Internals\\P5 G2 E>](file:///F:\Food%20allergy%20&%20intolerance\Papers\Paper%201%20submission\PLOSone\PLOSONE%20resubmission\a051d7cb-be9c-4186-bcd1-781060f3071b) -

Because they don’t know what’s in it…..With someone frowning going…’hmmm…no I don’t think we use gluten here’. So they obviously don’t know what it is. So that doesn’t instil me with hope.

And I don’t know if it’s a language thing…but the (Asian restaurant) they don’t know what you’re talking about. They’re very friendly and will cook you something but they don’t really know what you’re talking about.

[<Internals\\P6 G1 S>](file:///F:\Food%20allergy%20&%20intolerance\Papers\Paper%201%20submission\PLOSone\PLOSONE%20resubmission\c86011b3-5347-424b-84d1-781079ecb721)

even if it’s a certain type of sponge, or something, or something (child’s name) would quite like tonight, we ask the question, but if the staff are unsure we just don’t bother, which is a shame, because you think the staff should be more up to speed.

[<Internals\\P7 G3 NI>](file:///F:\Food%20allergy%20&%20intolerance\Papers\Paper%201%20submission\PLOSone\PLOSONE%20resubmission\080c88a7-da83-496b-add1-78109b8e897f)

Maybe when they’ve come and it’s – if something he’s chosen has come with cheese or something like that and you’ve asked for it not to have the cheese on it and they’ve brung it out with the cheese, then you would have to say look he can’t take the cheese or whatever.

[<Internals\\P8 G1 S>](file:///F:\Food%20allergy%20&%20intolerance\Papers\Paper%201%20submission\PLOSone\PLOSONE%20resubmission\49df7c58-716a-476d-bed1-7810c8a4f9c9)

You get a feeling sometimes when you go into a place and, to be honest, if people can’t reassure you we’re not going to sit and think well it might be okay, it might not. If they can’t, or if they take the liberty, of course, yes, its fine, then you think well you don’t really know what you’re talking about. You have to trust your gut instinct.

[<Internals\\P9 G1 NI>](file:///F:\Food%20allergy%20&%20intolerance\Papers\Paper%201%20submission\PLOSone\PLOSONE%20resubmission\45f12a10-9e9e-4aba-82d1-7810e482f550)

Yes. He has a dairy allergy and if there’s anything that has milk, cream or butter I will pick something different for him. We’ve been lied to a couple of times and my partner’s chased it straight away and we’ve literally said, you know, we’re not stupid….sometimes it happens and they intentionally do it because they don’t have time, so it does make things awkward, especially when you get into arguments with staff, ‘no it doesn’t’, ‘yes it does’. That has happened before, we have been in an argument with a manager of a restaurant because there’s milk in it, and cream, and a lot of salt. You just kind of have to be like, you know, this is going to have a really serious affect later.

.
